# ﻿A new four-pored *Amphisbaena* Linnaeus, 1758 (Amphisbaenia, Amphisbaenidae) from the north of Espinhaço Mountain Range, Brazil

**DOI:** 10.3897/zookeys.1213.122265

**Published:** 2024-09-24

**Authors:** Síria Ribeiro, Alfredo P. Santos Jr, Isabelly G. Martins, Elaine C. S. Oliveira, Roberta Graboski, Thiago Barbosa Da Silveira, Matheus H. M. Benício, Wilian Vaz-Silva

**Affiliations:** 1 Universidade Federal do Oeste do Pará, Programa de Pós-Graduação em Biodiversidade, 68040-255, Santarém, Pará, Brazil; 2 Laboratório de Ecologia e Comportamento Animal, Universidade Federal do Oeste do Pará, 68040-255, Santarém, Pará, Brazil; 3 Instituto Chico Mendes de Conservação da Biodiversidade, Coordenação Regional Oeste do Pará, 68040-000, Santarém, Pará, Brazil; 4 School of Zoology, Faculty of Life Sciences, the Steinhardt Museum of Natural History, Tel-Aviv University, Tel-Aviv, Israel; 5 Tecsan – Tecnologia e Saneamento Ltda – Rua Dr. José Peroba, 275, Ed. Metrópolis Empresarial, Sala 201/202, Stiep, 41770-235, Salvador, Bahia, Brazil; 6 Bahia Mineração S/A, Rodovia BA 156, s/n, Projeto Pedra de Ferro, 46400-000, Caetité, Bahia, Brazil; 7 Pontifícia Universidade Católica de Goiás, Escola de Ciências Médicas e da Vida, Centro de Estudos e Pesquisas Biológicas, and Programa de Pós-Graduação em Ciências Ambientais e Saúde, 74605-010, Goiânia, Goiás, Brazil

**Keywords:** Morphology, new species, phylogeny, taxonomy

## Abstract

A new species of *Amphisbaena* is described from the north of Espinhaço Mountain Range, municipality of Caetité, state of Bahia, Brazil. *Amphisbaenaamethysta***sp. nov.** can be distinguished from its congeners by the following combination of characters: (1) snout convex in profile, slightly compressed not keeled; (2) pectoral scales arranged in regular annuli; (3) four precloacal pores; (4) distinct cephalic shields; (5) 185–199 dorsal half-annuli; (6) 13–16 caudal annuli; (7) conspicuous autotomic site between 4^th^–6^th^ caudal annuli; (8) 16–21 dorsal and ventral segments at midbody; (9) 3/3 supralabials; (10) 3/3 infralabials; and (11) smooth and rounded tail tip. The new species is the 71^st^ species of genus with four precloacal pores, and the 22^nd^ species from the Caatinga morphoclimatic domain. The identification of *Amphisbaenaamethysta***sp. nov.** indicates that the reptile fossorial fauna in the Espinhaço Mountain Range region is far from being completely known and that it may harbour a much greater diversity of endemic taxa.

## ﻿Introduction

In the Caatinga 21 species of *Amphisbaena* have been recorded to date, with most being fully restricted to this morphoclimatic domain (Ribeiro and Eleutério 2023). Of these, four have wide distributions in Caatinga: *A.alba* Linnaeus, 1758, *A.lumbricalis* Vanzolini, 1996, *A.pretrei* Duméril & Bibron, 1839, and *A.vermicularis* Wagler, 1824, while nine have been found in a small number of localities: *A.anomala* (Barbour, 1914), *A.arenaria* Vanzolini, 1991, *A.bahiana* Vanzolini, 1964, *A.carvalhoi* Gans, 1965, *A.frontalis* Vanzolini, 1991, *A.fuliginosa* Linnaeus, 1758, *A.hastata* Vanzolini, 1991, *A.heathi* Schmidt, 1936, and *A.ignatiana* Vanzolini, 1991. In addition, five species are known only from the type locality: *A.arda* Rodrigues, 2003, *A.caetitensis* Almeida, Freitas, Silva, Valverde, Rodrigues, Pires & Mott, 2018, *A.longinqua* Teixeira Junior, Dal Vechio, Recoder, Cassimiro, Sena & Rodrigues, 2019, *A.mongoyo* Teixeira Junior, Dal Vechio, Recoder, Cassimiro, Sena & Rodrigues, 2019, and *A.uroxena* Mott, Rodrigues, Freitas & Silva, 2008 (Vanzolini 1992; Teixeira Jr et al. 2014; Costa et al. 2015; Almeida et al. 2018; Ribeiro and Eleutério 2023).

In the last two decades, six species of *Amphisbaena* have been identified in the high-altitude areas of the Espinhaço Mountain Range in Bahia and Minas Gerais: *A.bahiana*, *A.longinqua*, *A.metallurga* Costa, Resende, Teixeira Jr, Dal Vechio & Clemente, 2015, *A.mongoyo*, *A.uroxena*, and *A.caetitensis* (Costa et al. 2015; Almeida et al. 2018; Teixeira Junior et al. 2019). Four of which are known to be closely related phylogenetically (Teixeira Junior et al. 2019), while the position of the *A.metallurga* awaits analysis; and that of *A.caetitensis* awaits resolution of the phylogenetic relationship (Almeida et al. 2018). Such clustering points to a probable centre of endemism for this type of geological formation.

During a faunal rescue in the south of state of Bahia, Brazil, conducted as part of environmental activities carried by BAMIN (Bahia Mineração mining company), specimens of a species of *Amphisbaena* with four precloacal pores were collected, which could not be identified as belonging to any known amphisbaenid species. Accordingly, it was concluded that they represented a new taxon, which is described below.

## ﻿Materials and methods

We analysed 48 specimens of non-identified *Amphisbaena* from the municipality of Caetité, state of Bahia, Brazil. The type series was deposited in the herpetological collection from Pontifícia Universidade Católica de Goiás (CEPB), municipality of Goiânia, state of Goiás, Brazil. For morphological comparisons we used data from 370 analysed specimens of *Amphisbaena* (see list SI1). Additional morphological data was taken from the literature. The taxonomy follows the nomenclature of Guedes et al. (2023) for species (but not including subspecies). Nomenclature for the cephalic scales and for meristic data follow Gans and Alexander (1962). Morphometric data follow the methodology of Perez et al. (2012). Head scale morphometric data were taken with digital callipers (precision 0.01 mm) on the right side of specimens. Head length was measured from the tip of the rostral shield to the anterior margin of the first dorsal half-annulus; and ventral length of head was measured from tip of rostral shield to the anterior margin of first body annulus. Snout-vent length was measured with the aid of a nylon line and subsequently measured with a millimetre rule. Counts for dorsal and ventral half-annuli were made on the right side of each specimen. Incomplete half-annuli were not included in the total count. Observations on specimen and annuli counts were made with the aid of a stereomicroscope. Infralabials, supralabials, and parietal shields variations are treated as “right/left”.

Our molecular data matrix comprises 81 terminals for six genes: three mitochondrial genes (16S– 16S Large Subunit Ribosomal RNA gene; 12S – Small Subunit Ribosomal RNA gene; and *nd2* – NADH Dehydrogenase 2 gene) and three nuclear genes (*c-mos* – Oocyte maturation factor Mos; *bdnf* – Brain-derived neurotrophic factor; and *rag1* – Recombinant activating gene 1). We sequenced four new DNA fragments (two for 12S and two for 16S) for two specimens of *Amphisbaenaamethysta* sp. nov. (see Suppl. material 1: table S1). We also included sequences available in GenBank (https://www.ncbi.nlm.nih.gov) for 79 species of Amphisbaenia. We rooted our phylogenetic tree using Lacertidae (*Lacertamedia* Lantz & Cyrén, 1920).

DNA was extracted from liver tissue using the PureLink extraction kit (Invitrogen, Massachusetts, USA), following the manufacturer’s protocol. Sequences were amplified by Polymerase Chain Reaction (PCR) using the primers 12S and 16S as described by Graboski et al. (2023) following the amplification protocols described in Kearney and Stuart (2004) and Mott and Vieites (2009). Amplified fragments were purified with shrimp alkaline phosphatase and exonuclease I (GE Healthcare, Piscataway, NJ, USA). Both strands were sequenced on an Applied Biosystems 3500 Series Genetic Analyzer (Thermo Fisher Scientific, USA) at Laboratório de Genética e Biodiversidade da Universidade Federal de Goiás, Brazil. Both strands were quality-checked and, when necessary, edited manually. Consensus for both strands was generated using Geneious Prime 2022.1.1 (https://www.geneious.com).

Sequences were aligned using MAFFT 1.3.6 (Katoh and Standley 2013) through a plugin implemented in Geneious Prime. The 16S and 12S sequences were aligned under the E-INS-I algorithm, while nd2 and nuclear genes were aligned under the G-INS-i algorithm. We used default parameters for gap opening and extension. The protein-coding gene alignments were visually checked using Geneious Prime to verify that all sequences follow the correct reading frame. All genes were concatenated using Geneious Prime.

We used PartitionFinder 2 (Lanfear et al. 2016) to identify the combined best-fitting of partitioning schemes and models of molecular evolution. Our input matrix was divided in 14 partitions (coding genes were partitioned by codon positions and each rRNA was analysed as a separate partition) and was analysed using the greedy option. We performed a run allowing the program to select (using the Akaike Information Criterion with correction: AIC) for molecular evolution models implemented on RAxML (models GTR and GTR+G). We performed a maximum likelihood (ML) analysis using RAxML 8.2.3 (Stamatakis 2014). The ML tree was estimated using the RAxML algorithm that conducts a rapid bootstrap analysis and searches for best scoring ML tree in the same run (option -f a). We ran 1000 bootstrap replicates, and the best scoring ML tree was estimated 200 times using as a starting tree each fifth bootstrap tree. We also calculate uncorrected genetic distance (p-distance) using PAUP 4.0 (Swofford 2003). We considered only bootstrap values above 70% as a strong support.

## ﻿Results

### ﻿ Amphisbaena amethysta sp. nov.

Taxon classificationAnimaliaSquamataAmphisbaenidae

697AF1B8-7B5E-5EFA-BEF6-02ADD4E48B4D

https://zoobank.org/E34029C4-6E1A-4E87-89C7-99E70AC1B95A

Figs 1 , 2 , 3 , 4

#### Type material.

***Holotype***: • male; CEPB 2311; municipality of Caetité, state of Bahia, Brazil; [14°21'31"S, 42°32'19"W; 1012 m above sea level (a.s.l.)]; collected on 1 November 2022 by Faunal Rescue Team Tecsam (F. Santos, P. Belufi, and G. Nascimento). ***Paratypes***: • All from Caetité, Bahia, Brazil; collected by Faunal Rescue Team Tecsam (R. Assunção, A. Hirota, T. Silveira, F. Santos, P. Belufi, and G. Nascimento) • Female; CEPB 2301; (14°21'53"S, 42°32'20"W; 1013 m a.s.l.); 8 June 2022 • Male; CEPB 2302; (14°01'16"S, 42°31'06"W; 1082 m a.s.l.); 13 October 2021 • Female; CEPB 2303; (14°21'50"S, 42°32'20"W; 1013 m a.s.l.); 26 May 2022 • Female; CEPB 2308; (14°19'49"S, 42°32'44"W; 1011 m a.s.l.); 8 September 2022 • Female; CEPB 2327; (14°19'52"S, 42°32'42"W; 1011 m a.s.l.); 1 October 2022 • Female; CEPB 2331; (14°19'45"S, 42°32'45"W; 1011 m a.s.l.); 30 August 2022 • Male; CEPB 2346; (14°22'08"S, 42°32'17"W; 923 m a.s.l.); 6 October 2022 • Male; CEPB 2379; (14°21'27"S, 42°32'20"W; 1033 m a.s.l.); 26 January 2023 • Female; CEPB 2381; (14°21'27"S, 42°32'20"W; 1033 m a.s.l.); 26 January 2023.

#### Referred specimens.

All from Caetité, Bahia, Brazil; collected by Faunal Rescue Team Tecsam (R. Assunção, A. Hirota, T. Silveira, F. Santos, P. Belufi, and G. Nascimento) • Female; CEPB 2298; (14°21'53"S, 42°32'20"W; 1013 m a.s.l.); 8 June 2022 • Female; CEPB 2299; (14°21'53"S, 42°32'20"W; 1013 m a.s.l.); 8 June 2022 • Male; CEPB 2300; (14°21'51"S, 42°32'21"W; 1011 m a.s.l.); 25 May 2022 • Female; CEPB 2304; (14°21'53"S, 42°32'20"W; 1013 m a.s.l.); 8 June 2022 • Female; CEPB 2305; (14°21'53"S, 42°32'20"W; 1013 m a.s.l.); 8 June 2022 • Female; CEPB 2306; (14°21'53"S, 42°32'20"W; 1013 m a.s.l.); 13 August 2022 • Male; CEPB 2307; (14°21'23"S, 43°32'07"W; 972 m a.s.l.); 23 November 2022 • Undetermined sex; CEPB 2309; (14 °19'46"S, 42°43'20"W; 1011 m a.s.l.); 1 September 2022 • Female; CEPB 2310; (14°19'55"S, 42°32'44"W; 1011 m a.s.l.); 8 October 2022 • Male; CEPB 2312; (14°19'56"S, 42°32'43"W; 1011 m a.s.l.); 12 October 2022 • Female; CEPB 2313; (14°22'08"S, 42°32'17"W; 9215 m a.s.l.); 6 October 2022 • Female; CEPB 2314; (14°22'08"S, 42°32'17"W; 925 m a.s.l.); 6 October 2022 • Female; CEPB 2315; (14°22'08"S, 42°32'17"W; 925 m a.s.l.); 6 October 2022 • Female; CEPB 2316; (14°19'47"S, 42°32'43"W; 1011 m a.s.l.); 1 September 2022 • Female; CEPB 2317; (14°19'55"S, 43°32'44"W; 1011 m a.s.l.); 1 October 2022 • Female; CEPB 2318; (14°19'46"S, 42°32'43"W; 1011 m a.s.l.); 1 September 2022 • Female; CEPB 2319; (14°20'37"S, 42°32'13"W; 1076 m a.s.l.); 3 August 2022 • Female; CEPB 2320; (14°20'38"S, 42°32'12"W; 1013 m a.s.l.); 2 June 2022 • Female; CEPB 2321; (14°19'53"S, 42°32'43"W; 1011 m a.s.l.); 29 September 2022 • Female; CEPB 2322; (14°14'28"S, 42°32'47"W; 842 m a.s.l.); 17 September 2022 • Female; CEPB 2323; (14°19'49"S, 42°32'17"W; 1062 m a.s.l.); 13 August 2022 • Male; CEPB 2324; (14°19'48"S, 42°32'43"W; 1011 m a.s.l.); 6 September 2022 • Female; CEPB 2325; (14°19'55"S, 42°32'44"W; 1011 m a.s.l.); 1 October 2022 • Male; CEPB 2326; (14°20'52"S, 43°32'16"W; 1053 m a.s.l.); 13 August 2022 • Female; CEPB 2328; (14°21'53"S, 42°32'22"W; 1011 m a.s.l.); 28 September 2022 • Male; CEPB 2329; (14°19'47"S, 42°32'43"W; 1011 m a.s.l.); 1 September 2022 • Male; CEPB 2330; (14°19'55"S, 43°32'44"W; 1011 m a.s.l.); 1 October 2022 • Male; CEPB 2332; (14°20'50"S, 42°32'16"W; 1057 m a.s.l.); 13 August 2022 • Female; CEPB 2333; (14°14'28"S, 42°32'47"W; 842 m a.s.l.); 17 September 2022 • Female; CEPB 2334; (14°19'46"S, 42°32'43"W; 1011 m a.s.l.); 1 September 2022 • Female; CEPB 2336; (14°15'11"S, 45°32'16"W; 987 m a.s.l.); 3 August 2022 • Female; CEPB 2337; (14°20'38"S, 45°32'12"W; 1076 m a.s.l.); 3 August 2022 • Female; CEPB 2338; (14°21'31"S, 45°32'16"W; 1010 m a.s.l.); 13 August 2022 • Female; CEPB 2339; (14°14'28"S, 42°32'47"W; 842 m a.s.l.); 17 September 2022 • Male; CEPB 2356; (14°21'27"S, 42°32'21"W; 1033 m a.s.l.); 1 November 2022 • Male; CEPB 2379; (14°21'27"S, 42°32'20"W; 1033 m a.s.l.); 26 January 2023 • Female; CEPB 2380; (14°21'27"S, 42°32'20"W; 1033 m a.s.l.); 26 January 2023 • Female; CEPB 2381; (14°21'27"S, 42°32'20"W; 1033 m a.s.l.); 26 January 2023.

#### Diagnosis and comparisons with other south American amphisbaenians.

*Amphisbaenaamethysta* sp. nov. is a medium-sized amphisbaenid (258 mm maximum snout-vent length), and can be distinguished from its congeners by the following combination of characters (see details in Table 1): (1) snout convex in profile view, slightly compressed not keeled; (2) pectoral scales arranged in regular annuli; (3) four precloacal pores; (4) distinct cephalic shields; (5) 185–199 dorsal half-annuli; (6) 13–16 caudal annuli; (7) conspicuous autotomic site between 4^th^–6^th^ caudal annuli; (8) 16–21 dorsal and ventral segments at midbody; (9) 3/3 supralabials; (10) 3/3 infralabials; and (11) smooth and rounded tail tip.

*Amphisbaenaamethysta* sp. nov. differs from *Amphisbaenaacrobeles* (Ribeiro, Castro-Mello & Nogueira, 2009), *A.bilabialata* (Stimson, 1972), *A.kingi* (Bell, 1833), *A.anomala*, *Mesobaenahuebneri* Mertens, 1925; *M.rhachicephala* Hoogmoed, Pinto, Rocha & Pereira, 2009; and all *Leposternon* species, mainly in having the snout convex in profile view, slightly compressed not keeled (*vs* snout hardly compressed forming a sharp and prominent keel or snout depressed shovel-like). Differs still from *A.anomala* and all *Leposternon* species by having pectoral scales arranged in regular annuli (*vs* pectoral scales with an irregular form, and dermal annuli not regularly arranged).

*Amphisbaenaamethysta* sp. nov. presents four precloacal pores, differing from all *Amphisbaena* species except *A.acangaoba* Ribeiro, Gomides & Costa, 2020, *A.alba*, *A.albocingulata* Boettger, 1885, *A.angustifrons* Cope, 1861, *A.arda*, *A.arenaria*, *A.arenicola* Perez & Borges-Martins, 2019, *A.bahiana*, *A.bakeri* Stejneger, 1904, *A.barbouri* Gans & Alexander, 1962, *A.bedai* (Vanzolini, 1991), *A.bolivica* Mertens, 1929, *A.borellii* Peracca, 1897, *A.brasiliana* (Gray, 1865), *A.caeca* Cuvier, 1829, *A.camura* Cope, 1862, *A.carlgansi* Thomas & Hedges, 1998, *A.carioca* Rocha, Barros-Filho & Sluys, 2023, *A.carvalhoi*, *A.caudalis* Cochron, 1928, *A.cayemite* Thomas & Hedges, 2006, *A.cegei* Montero, Sáfadez, Álvarez, 1997, *A.cubana* Gundlach & Peters, 1879, *A.cuiabana* (Strüssmann & Carvalho, 2001), *A.cunhai* Hoogmoed & Ávila-Pires, 1991, *A.darwini*, *A.elbakyanae* Torres-Ramírez, Angarita-Sierra & Vargas-Ramírez, 2021, *A.fenestrata* (Cope, 1861), *A.frontalis*, *A.gonavensis* Gans & Alexander, 1962, *A.gracilis* Strauch, 1881, *A.hastata*, *A.heathi*, *A.hogei* Vanzolini, 1950, *A.hoogmoedi* Oliveira, Vaz-Silva, Santos-Jr, Graboski, Teixeira Jr, Dal Vechio & Ribeiro, 2018, *A.hyporissor* Thomas, 1965, *A.innocens* Weinland, 1862, *A.kingi*, *A.kraoh* (Vanzolini, 1971), *A.leali* Thomas & Hedges, 2006, *A.lumbricalis*, *A.manni* Barbour, 1914, *A.medemi* Gans & Mathers, 1977, *A.metallurga*, *A.mongoyo*, *A.munoai* Klappenbach, 1960, *A.myersi* Hoogmoed, 1989, *A.nana* Perez & Borges-Martins, 2019, *A.nigricauda* Gans, 1966, *A.occidentalis* Cope, 1875, *A.pericensis* Noble, 1921, *A.plumbea* Gray, 1872, *A.polygrammica* Werner, 1901, *A.prunicolor* (Cope, 1885), *A.ridleyi* Boulenger, 1890, *A.rozei* Lancini, 1963, *A.sanctaeritae* Vanzolini, 1994, *A.saxosa* (Castro-Mello, 2003), *A.schmidti* Gans, 1964, *A.slateri* Boulenger, 1907, *A.slevini* Schmidt, 1936, *A.spurrelli* Boulenger, 1915, *A.steindachneri* Strauch, 1881, *A.supernumeraria* Mott, Rodrigues & Dos Santos, 2009, *A.talisiae* Vanzolini, 1995, *A.tyaraju*, Perez & Borges-Martins, 2019, *A.townsendi* Stejneger, 1911, *A.tragorrhectes* Vanzolini, 1971, *A.uroxena*, *A.vanzolinii* Gans, 1963, *A.vermicularis*, and *A.xera* Thomas, 1966.

*Amphisbaenaamethysta* sp. nov. differs from *Amphisbaena* species with four precloacal pores mainly by following combination of meristic characters (Table 1): cephalic shield distinct (vs frontals and parietals shields not distinct in *A.supernumeraria*, ocular and second supralabial not distinct in *A.cubana*); snout slightly compressed (vs hard compressed in *A.kingi* and rounded in all other species, except *A.kraoh*, *A.brasiliana*, *A.bahiana*, *A.bedai*, and *A.saxosa*); 185–199 dorsal half-annuli (vs < 170 annuli in *A.cayemite* and > 200 annuli in *A.acangaoba*, *A.arda*, *A.arenaria*, *A.bahiana*, *A.bakeri*, *A.barbouri*, *A.bedai*, *A.borellii*, *A.brasiliana*, *A.caeca*, *A.carlgansi*, *A.carvalhoi*, *A.cayemite*, *A.cuiabana*, *A.cunhai*, *A.elbakyanae*, *A.fenestrata*, *A.frontalis*, *A.gonavensis*, *A.gracilis*, *A.hastata*, *A.hoogmoedi*, *A.kingi*, *A.kraoh*, *A.lumbricalis*, *A.manni*, *A.medemi*, *A.mongoyo*, *A.munoai*, *A.myersi*, *A.nigricauda*, *A.occidentalis*, *A.plumbea*, *A.polygrammica*, *A.rozei*, *A.sanctaeritae*, *A.saxosa*, *A.slevini*, *A.spurrelli*, *A.steindachneri*, *A.supernumeraria*, *A.talisiae*, *A.tyaraju*, *A.townsendi*, *A.uroxena*, *A.vanzolinii*, *A.vermicularis*, and *A.xera*); 13–16 caudal annuli (vs > 16 annuli in *A.albocingulata*, *A.arda*, *A.arenaria*, *A.arenicola*, *A.bedai*, *A.bolivica*, *A.borellii*, *A.carvalhoi*, *A.caudalis*, *A.cegei*, *A.cuiabana*, *A.cunhai*, *A.darwini*, *A.elbakyanae*, *A.frontalis*, *A.gracilis*, *A.hastata*, *A.hoogmoedi*, *A.leali*, *A.lumbricalis*, *A.manni*, *A.medemi*, *A.metallurga*, *A.mongoyo*, *A.munoai*, *A.myersi*, *A.nana*, *A.nigricauda*, *A.occidentalis*, *A.polygrammica*, *A.prunicolor*, *A.rozei*, *A.sanctaeritae*, *A.saxosa*, *A.schmidti*, *A.slateri*, *A.slevini*, *A.spurrelli*, *A.steindachneri*, *A.supernumeraria*, *A.talisiae*, *A.tyaraju*, *A.townsendi*, *A.tragorrhectes*, and *A.vermicularis*); conspicuous autotomic site between 4^th^–6^th^ caudal annuli (vs absent in *A.acangaoba*, *A.alba*, *A.angustifrons*, *A.bakeri*, *A.barbouri*, *A.brasiliana*, *A.carioca*, *A.carlgansi*, *A.cayemite*, *A.cunhai*, *A.fenestrata*, *A.gonavensis*, *A.hastata*, *A.hoogmoedi*, *A.innocens*, *A.lumbricalis*, *A.occidentalis*, *A.ridleyi*, *A.saxosa*, and *A.uroxena*; or from the 7^th^ caudal annuli in *A.albocingulata*, *A.arda*, *A.arenicola*, *A.carvalhoi*, *A.cegei*, *A.cuiabana*, *A.darwini*, *A.heathi*, *A.kingi*, *A.metallurga*, *A.mongoyo*, *A.myersi*, *A.nana*, *A.prunicolor*, *A.schmidti*, *A.slateri*, *A.steindachneri*, *A.supernumeraria*, *A.talisiae*, *A.tyaraju*, *A.townsendi*, *A.tragorrhectes*, and *A.vanzolinii*), smooth and rounded tail tip [vs bluntly ridged tail tip in *A.bahiana*; slightly dorsally compressed in *A.acangaoba*; with tubercles sit is depressed (compressed dorsoventrally) in *A.uroxena*; with modified conic pointed tubercles in *A.caetitensis*; and vertically keeled in *A.borellii* and *A.steindachneri*]; 16–21 dorsal segments at midbody (vs < 16 in *A.albocingulata*, *A.arenaria*, *A.arenicola*, *A.barbouri*, *A.carlgansi*, *A.carvalhoi*, *A.cayemite*, *A.cuiabana*, *A.elbakyanae*, *A.fenestrata*, *A.heathi*, *A.hogei*, *A.elbakyanae*, *A.metallurga*, *A.munoai*, *A.nana*, *A.nigricauda*, *A.sanctaeritae*, *A.schmidti*, *A.slateri*, *A.slevini*, *A.supernumeraria*, *A.talisiae*, *A.tyaraju*, *A.tragorrhectes*, *A.uroxena*, and *A.vanzolinii*); and > 21 segments in *A.alba*, *A.arda*, *A.bolivica*, *A.camura*, *A.hoogmoedi*, and *A.kraoh*); 16–21 ventral segments at midbody (vs < 16 in *A.rozei*, *A.sanctaeritae*, and *A.tragorrhectes*; and > 21 segments in *A.alba*, *A.arda*, *A.bolivica*, *A.camura*, *A.cegei*, *A.gonavensis*, *A.hyporissor*, *A.kraoh*, *A.occidentalis*, and *A.townsendi*); 3/3 supralabials (vs 2/2 in *A.slevini* and *A.vanzolinii*; and 4/4 in *A.acangaoba*, *A.alba*, *A.angustifrons*, *A.arda*, *A.arenaria*, *A.bedai*, *A.camura*, *A.cayemite*, *A.occidentalis*, *A.plumbea*, *A.ridleyi*, *A.saxosa*, *A.townsendi*, *A.tragorrhectes*, and *A.vermicularis*); and 3/3 infralabials (vs 2/2 in *A.slevini* and *A.vanzolinii*; and or 4/4 in *A.occidentalis*, *A.plumbea*, *A.ridleyi*, *A.saxosa*, *A.townsendi*, and *A.tragorrhectes*).

**Table 1.** Diagnostic characters for the *Amphisbaena* species with four precloacal pores. PC – precloacal pore, CS – cephalic shield, DA – dorsal half-annulus, CA – caudal annulus, AS – autotomic site, DS – dorsal segment, VS – ventral segment, SL – supralabial, IL – infralabial, PM – postmalar, SN – snout, DG – dorsal groove, VG – ventral groove, a – absent, cp – compressed, n/a – non-available data, p – present, rd – rounded, and sc – slightly compressed. Differences from the new species in bold.

Species	PC	CS	DA	CA	AS	DS	VS	SL	IL	PM	SN	DG	VG	Reference
*A.amethysta* sp. nov.	4	distinct and paired	185–199	13–16	4–6	16–21	16–21	3	3	a	sc	a	a	present study
*A.acangaoba*	4–8	distinct and paired	**216–293**	13–17	**a**	18–24	18–24	**4**	3	**p**	**rd**	a	a	Ribeiro et al. (2020)
*A.alba*	4–10	distinct and paired	198–248	13–21	**a**	**30–42**	**35–46**	**4**	3	**p**	**rd**	a	a	Gans (1962a)
*A.albocingulata*	4	distinct and paired	190–204	**24–27**	**8–9**	**12–14**	16–18	3	3	a	**rd**	a	a	Perez et al. (2012)
*A.angustifrons*	3–6	distinct and paired	190–215	12–18	**a**	20–31	21–30	**4**	3	**p**	**rd**	a	a	Gans (1965a); Gans and Diefenbach (1972)
*A.arda*	4	distinct and paired	**242**	**30**	**8**	**23**	**23**	**4**	3	**p**	**rd**	a	a	Rodrigues et al. (2003)
*A.arenaria*	4	distinct and paired	**285–307**	**22–23**	6–7	**12–14**	14–16	**4**	3	a	**rd**	a	a	Teixeira Junior et al. (2016)
*A.arenicola*	4	distinct and paired	199–216	**20–22**	**8–9**	**12–14**	16–18	3	3	**P**	**rd**	n/a	n/a	Perez and Borges-Martins (2019)
*A.bahiana*	4	distinct and paired	**204–223**	14–16	4–5	12–16	14–16	3	3	**p**	sc	**p**	**p**	Gans (1964b); Dal Vechio et al. (2018)
*A.bakeri*	4	distinct and paired	**239–255**	14–16	**a**	14–16	16–17	3	3	a	**rd**	n/a	n/a	Gans and Alexander (1962)
*A.barbouri*	4–6	distinct and paired	**226–240**	13–18	**a**	**12–14**	16–18	3	3	a	**rd**	n/a	n/a	Gans and Alexander (1962); Thomas (1966)
*A.bedai*	4	distinct and paired	**272–284**	**22–23**	6	18–20	16–18	**4**	3	**p**	sc	a	a	Oliveira et al. (2018)
*A.bolivica*	4–6	distinct and paired	200–231	**18–26**	4–5	**27–36**	**26–36**	3	3	**p**	**rd**	a	a	Montero (1996)
*A.borellii*	4	distinct and paired	**239–245**	**17–19**	6–8	14–16	16–20	3	3	n/a	**rd**	a	a	Gans (1964b) and Oliveira et al. (2018)
*A.brasiliana*	4	distinct and paired	**213–229**	11–15	**a**	18–21	18–22	3	3	**p**	sc	**P**	a	Oliveira et al. (2018)
*A.caeca*	4–6	distinct and paired	**217–236**	13–18	4–8	13–18	14–20	3	3	**p**	**rd**	n/a	n/a	Gans and Alexander (1962)
*A.caetitensis*	4	distinct and paired	**186–194**	**10–12**	**a**	16	19–22	3	3	a	**rd**	a	a	Almeida et al. (2018)
*A.camura*	4–6	distinct and paired	188–207	14–19	4–5	**28–42**	**29–46**	**4**	3	**p**	**rd**	a	a	Gans (1965a); Hoogmoed and Ávila-Pires (1991)
*A.carioca*	4	distinct and paired	186	**21**	**a**	**10–12**	n/a	3	3	**p**	**rd**	a	a	Rocha et al. (2023)
*A.carlgansi*	4	distinct and paired	**212–228**	14–16	**a**	**14**	18–20	3	3	a	**rd**	a	a	Thomas and Hedges (1998)
*A.carvalhoi*	4	distinct and paired	**231–245**	**19–22**	**7–8**	**12–14**	16–18	3	3	a	**rd**	a	a	Gans (1965b)
*A.caudalis*	4	distinct and paired	193–217	**17–21**	6	12–16	18–21	3	3	a	**rd**	n/a	n/a	Gans and Alexander (1962); Thomas and Hedges (1998)
*A.cayemite*	4	distinct and paired	**150–164**	10–13	**a**	**12–13**	18	**4**	3	**p**	**rd**	n/a	n/a	Thomas and Hedges (2006)
*A.cegei*	4	distinct and paired	198	**22**	**7**	21–22	**22–23**	3	3	a	**rd**	n/a	n/a	Montero et al. (1997)
*A.cubana*	4–6	**ocular and second SL fused**	199–218	10–16	6–9	12–16	14–18	3	3	a	**rd**	n/a	n/a	Gans and Alexander (1962); Thomas (1966)
*A.cuiabana*	4	distinct and paired	**286–292**	**18–20**	**9–10**	**14**	16	3	3	a	**rd**	**p**	a	Strüssmann and Carvalho (2001)
*A.cunhai*	4	distinct and paired	**226–239**	**25–26**	**a**	14–16	14–18	3	3	**p**	**rd**	a	a	Hoogmoed and Ávila-Pires (1991)
*A.darwini*	2–5	distinct and paired	174–195	**19–25**	**7–10**	13–19	16–23	3	3	n/a	**rd**	a	a	Perez et al. (2012)
*A.elbakyanae*	4	distinct and paired	**245–257**	**20–24**	6–7	**13–15**	16–18	3	3	**p**	**rd**	**p**	a	Torres-Ramírez et al. (2022)
*A.fenestrata*	4	distinct and paired	**236–249**	12–14	**a**	**13–14**	14–16	3	3	**p**	**rd**	n/a	n/a	Gans and Alexander (1962
*A.frontalis*	0–4	distinct and paired	**235–275**	**23–29**	5–7	14–18	14–16	3	3	a	**rd**	a	a	Ribeiro-Júnior et al. (2022)
*A.gonavensis*	4	distinct and paired	**214–225**	10–13	**a**	15–18	**22–25**	3	3	**p**	**rd**	n/a	n/a	Gans and Alexander (1962)
*A.gracilis*	4	distinct and paired	**224–248**	**21–24**	6–7	13–16	14–17	3	3	**p**	**rd**	**p**	a	Gonzales-Sponga and Gans (1971)
*A.hastata*	4	distinct and paired	**266–273**	**40**	**a**	18	16	3	3	a	**rd**	a	p	Vanzolini (1991)
*A.heathi*	4	distinct and paired	183–187	n/a	**7–8**	**12**	18–20	3	3	a	**rd**	a	a	Gans (1965b)
*A.hogei*	4	distinct and paired	177–191	15–19	4–7	**10–13**	14–18	3	3	**p**	**rd**	a	a	Gans (1966); Vanzolini (1950)
*A.hoogmoedi*	4	distinct and paired	**247–252**	**27**	**7–8**	**22–24**	**19–21**	3	3	a	sc	a	a	Oliveira et al. (2018)
*A.hyporissor*	4	distinct and paired	199–227	16–21	5	14–18	**22–24**	3	3	**p**	**rd**	n/a	n/a	Thomas (1965); Thomas and Hedges (2006)
*A.innocens*	4	distinct and paired	185–220	11–14	**a**	13–16	18–21	3	3	a	**rd**	n/a	n/a	Gans and Alexander (1962); Thomas and Hedges (1998)
*A.kingi*	4	distinct and paired	**214–244**	15–23	**7**	12–19	14–22	3	3	**p**	**cp**	a	a	Gans and Rhodes (1964); Oliveira et al. (2018)
*A.kraoh*	4–6	distinct and paired	**270–281**	15	5	**28**	**24–27**	3	3	n/a	sc	n/a	n/a	Oliveira et al. (2018)
*A.leali*	4	distinct and paired	188–206	**17–20**	6	14–16	20–22	3	3	a	**rd**	n/a	n/a	Thomas and Hedges (2006)
*A.lumbricalis*	2–6	distinct and paired	**225–247**	**20–26**	**a**	12–16	16–20	3	3	n/a	**rd**	a	a	Vanzolini (1996)
*A.manni*	4–9	distinct and paired	**209–243**	**17–22**	5–7	12–16	14–20	3	3	a	**rd**	n/a	n/a	Gans and Alexander (1962)
*A.medemi*	4	distinct and paired	**230–235**	**17–18**	5–7	16	18	3	3	a	**rd**	a	**p**	Gans and Mather (1977)
*A.metallurga*	2–4	distinct and paired	185–199	**23–25**	**7–9**	**12–14**	14–16	3	3	**p** or a	**rd**	a	a	Costa et al. (2019)
*A.mongoyo*	4	distinct and paired	**208**	**25**	**10**	**14**	16	3	3	a	**rd**	a	a	Teixeira Junior et al. (2019)
*A.munoai*	4	distinct and paired	**202–218**	**18–23**	5–9	**10–14**	13–18	3	3	**p**	**rd**	a	a	Perez and Borges-Martins (2019)
*A.myersi*	4	distinct and paired	**221**	**28**	**8**	16	16	3	3	n/a	**rd**	a	a	Hoogmoed (1989)
*A.nana*	4	distinct and paired	195–216	**18–22**	**7–10**	**12–14**	14–17	3	3	**p**	**rd**	n/a	n/a	Perez and Borges-Martins (2019)
*A.nigricauda*	4	distinct and paired	**222–226**	**19–24**	6–9	**10**	16	3	3	a	**rd**	a	a	Gans (1966)
*A.occidentalis*	4	distinct and paired	**261–279**	**18–21**	**a**	16–19	**22–27**	**4**	**4**	**p**	**rd**	**p**	**p**	Gans (1961)
*A.pericensis*	4	distinct and paired	198–218	16–19	6–8	12–16	16–20	3	3	a	**rd**	a	a	Gans (1963a)
*A.plumbea*	4	distinct and paired	**233–282**	16–21	5–9	18–27	20–30	**4**	**4**	**p**	**rd**	a	a	Gans and Diefenbach (1972)
*A.polygrammica*	4	distinct and paired	**270**	**20**	n/a	18	16	n/a	n/a	n/a	n/a	n/a	n/a	Vanzolini (2002)
*A.prunicolor*	4	distinct and paired	181–215	**18–27**	**7–10**	10–17	14–20	3	3	**p**	**rd**	a	a	Perez et al. (2012)
*A.ridleyi*	4	distinct and paired	172–192	14–17	**a**	16–18	20–28	**4**	**4**	**p**	**rd**	a	a	Gans (1963b)
*A.rozei*	4	distinct and paired	**205–209**	**20**	6 or a	15–16	**14**	3	3	**p**	**rd**	a	a	Vanzolini (2002); Costa et al. (2019)
*A.sanctaeritae*	4	distinct and paired	**269–288**	18–20	6–7	**10**	**14**	3	3	**p**	**rd**	a	a	Costa et al. (2019)
*A.saxosa*	4	distinct and paired	**253–272**	**17–21**	**a**	18–24	16–21	**4**	**4**	**p**	sc	a	a	Oliveira et al. (2018)
*A.schmidti*	4	distinct and paired	198–202	**20–22**	**7–8**	**14**	16–17	3	3	**p**	**rd**	a	a	Gans (1964a)
*A.slateri*	4	distinct and paired	176–213	**20–24**	**7–10**	**10–14**	14–16	3	3	**p** or a	**rd**	a	a	Gans (1967); Costa et al. (2018)
*A.slevini*	4	distinct and paired	**204–211**	**23–25**	5–6	**10–14**	10–12	**2**	**2**	a	**rd**	**p**	a	present study
*A.spurrelli*	4	distinct and paired	**218–222**	**18–20**	**6–7**	16–18	16–18	3	3	**p**	**rd**	a	a	Gans (1962b); Costa (2020)
*A.steindachneri*	4	distinct and paired	**256–266**	**17–18**	**7**	14–16	16	3	3	a	**rd**	**p**	**p**	Gans (1964b)
*A.supernumeraria*	4	**not distinct**	**333–337**	**22–23**	**10–12**	**14**	17–18	3	3	n/a	**rd**	a	n/a	Mott et al. (2009)
*A.talisiae*	4	distinct and paired	**205–234**	**17–29**	**7**	**10–14**	14–18	3	3	a	**rd**	a	a	Vanzolini (1995); Costa et al. (2019)
*A.tyaraju*	4	distinct and paired	**204–221**	**18–25**	**7–9**	**10–14**	13–16	3	3	**p**	**rd**	n/a	n/a	Perez and Borges-Martins (2019)
*A.townsendi*	4	distinct and paired	**261–279**	**22–26**	**7–8**	16–19	**22–27**	**4**	**4**	**p**	**rd**	n/a	n/a	Gans (1961)
*A.tragorrhectes*	4	distinct and paired	196	**31**	**12**	**12**	**12**	**4**	**4**	**p**	**rd**	a	a	present study
*A.uroxena*	0–4	distinct and paired	**210–213**	12–13	**a**	**14**	14–15	3	3	**p**	**rd**	a	**p**	Mott et al. (2008)
*A.vanzolinii*	4	distinct and paired	**225–228**	n/a	**7**	**12–13**	16–17	**2**	**2**	**p**	**rd**	a	a	Gans (1963c)
*A.vermicularis*	4	distinct and paired	**211–254**	**23–34**	5–7	18–26	18–25	**4**	3	n/a	**rd**	a	a	Gans and Amdur (1966)
*A.xera*	4	distinct and paired	**225–234**	13 –16	5	12–16	14–16	3	3	a	**rd**	a	a	Thomas (1966)

#### Description of the holotype.

**Figure 1.**
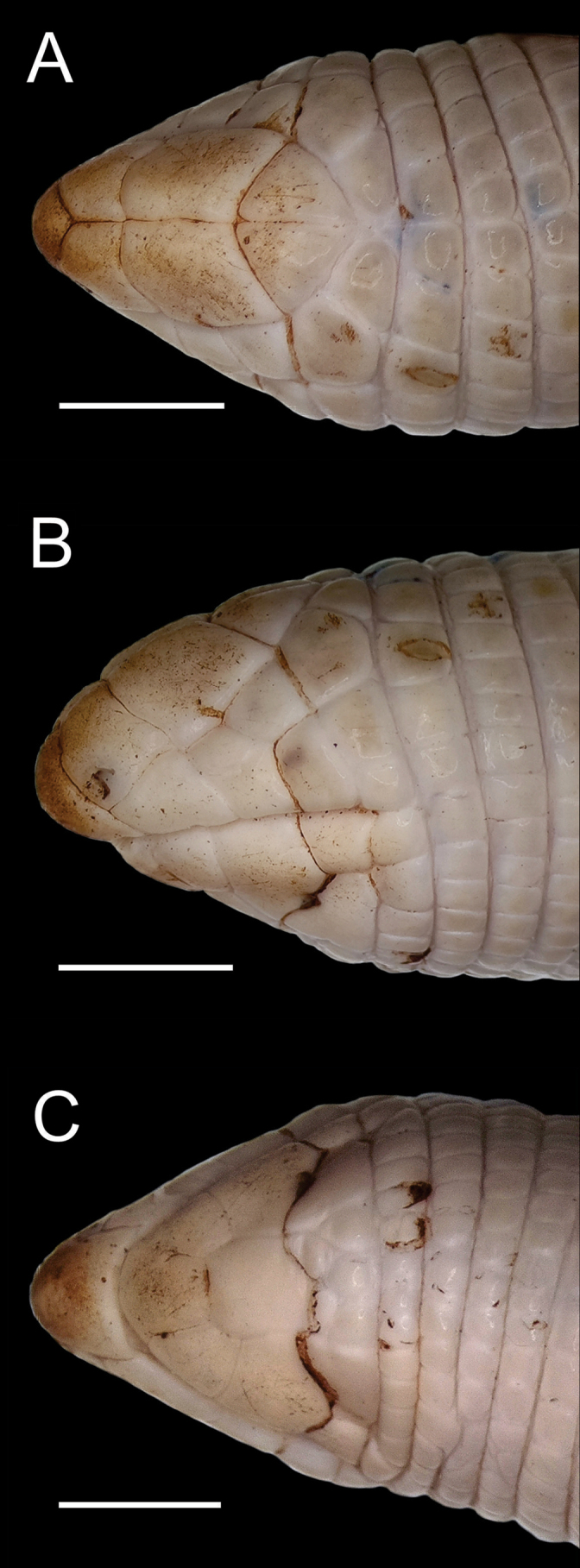
*Amphisbaenaamethysta* sp. nov. (Holotype, CEPB 2311) **A** dorsal **B** lateral, and **C** ventral views of head. Scale bar: 3 mm.

medium-sized specimen; snout-vent length 233 mm plus 0.50 mm of cloacal portion; tail length 21.24 mm, representing 9.1% of snout-vent length; midbody diameter 8.2 mm (3.5% of snout-vent length); head relatively small, 6.90 mm (~ 2.9% of snout-vent length); snout compressed in dorsal view and slightly convex in profile view, hardly keratinised, rostrum projecting forward beyond the jaw (prognathous). Anterior portion of body is slightly narrower. Rostral subtriangular, visible in dorsal and ventral view (Fig. 1), almost as high (2.21 mm) as wide (2.11 mm), in contact with nasal and first supralabial lateroposteriorly. Nasals subrectangular, aligned at the midline (1.00 mm suture) (Fig. 1A), almost as long (2.05 mm) as wide (1.99 mm), in contact with first supralabial laterally and prefrontals posteriorly, with nostrils placed near the antero-inferior angle of the nasal shield (Fig. 1B).

Prefrontals paired, relatively large (41.6% of head length), with a shorter middorsal suture (2.01 mm; 29.3% of head length), longer than the nasal middorsal suture (1.00 mm, 14.6% of head length), almost as long as frontal middorsal suture (2.10 mm, 30.6% of head length), anterior border convex, lateroposterior portion projected, in contact with second supralabial and ocular laterally, frontals posteriorly and in point contact with postocular posteriorly (Fig. 1A). Frontals subtriangular, longer (suture length) than wide (1.58 mm), aligned at midline (2.10 mm), in narrow contact with the oculars, and in broad contact posterolaterally with the postocular and parietal. Parietals in two larger irregulars segments, wider (1.48 mm) than long (1.04 mm), intercalated by four very smalls segments; not aligned at the midline, in narrow contact with postoculars laterally, and followed by the first dorsal half-annulus. Occipitals absent (Fig. 1A). Oculars almost diamond-shape, almost as long (1.57 mm) as high (1.51 mm), in contact with prefrontals and second supralabial anteriorly, third supralabial and postocular posteriorly, and in point contact with postsupralabial. Eyes slightly visible. Postocular longer than wide, sub-pentagonal, in contact with frontal, labial, parietal and the segments of the first dorsal half-annulus, and in point contact with prefrontal. Temporal subrectangular, higher (1.55 mm) than long (0.92 mm), in contact with third supralabial anteriorly, postocular and postsupralabial laterally and first dorsal half-annulus posteriorly (Fig. 1B).

Three supralabials, irregularly polygonal; first subtrapezoid, longer (2.05 mm) than high (1.50 mm), in contact with second supralabial posteriorly; second supralabial sub-pentagonal, higher (1.76 mm) than long (1.59 mm maximum length), in contact with prefrontal, ocular and third supralabial; third supralabial subtrapezoid, almost as high (1.37 mm) as long (1.24 mm), in contact with ocular and postsupralabial. Postsupralabial subquadrangular, representing almost 50% of third supralabial high, in contact with temporal laterally and first half-annulus posteriorly (Fig. 1B). Mental longer (1.72 mm) than wide, anterior border wider (1.72 mm) than posterior (1.08 mm), in contact with the first pair of infralabials and postmental. Postmental longer (2.01 mm) than wide (1.65 mm), in contact with the first and second infralabial, narrowly with malar, and two anterior postgenials. Postgenials with five shields irregularly distributed, in contact with malars and first ventral half-annuli (Fig. 1C).

Three infralabials, first medium sized, irregular polygonal, almost as long (1.55 mm) as wide (1.56 mm), in contact with second supralabial; second the largest, sub-pentagonal, wider (2.36 mm) than long (1.81 mm maximum length), in contact with third infralabial; third infralabial smallest, almost as long (1.24 mm) as wide (1.37 mm) (Fig. 1C).

**Figure 2.**
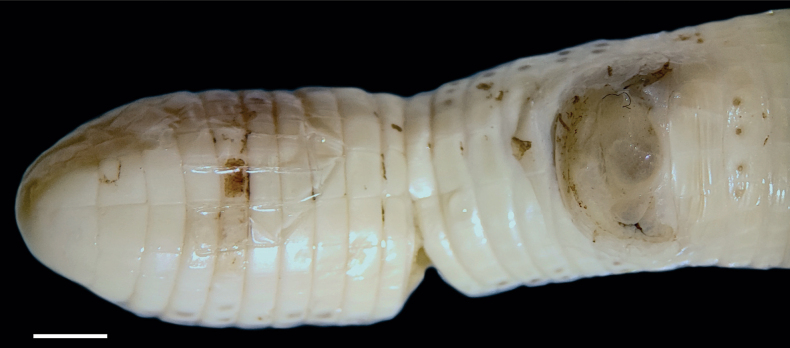
Tail of *Amphisbaenaamethysta* sp. nov. (Holotype, CEPB 2311). Detail of the autotomic site and four precloacal pores. Scale bar: 3 mm.

Body annuli well demarcated, first and second annuli without enlarged dorsal segments. Segments become regularly rectangular toward posterior portion of body and progressively longer than wide, and smaller in size, and larger towards midventral areas starting from the fifth half-annulus. One hundred ninety-four dorsal and 195 ventral half-annuli, three lateral half-annuli, 14 caudal annuli plus tip rounded; tail relatively long with autotomy line on the fifth annulus, 18/18 dorsal and ventral segments at midbody, respectively and 28 segments in fourth caudal annulus. Lateral sulci clearly visible from the forty-ninth annulus; dorsal and ventral sulci absent. Cloacal plate with six segments increasing in size from towards midline, eleven postcloacal segments; four precloacal pores strongly visible on the row of segments on the last ventral half-annulus; each pore placed on the posterior half of a single segment, and distributed along a continuous series of segments, but pores in the medial scales placed laterally (Fig. 2).

#### Intraspecific variation.

**Figure 3.**
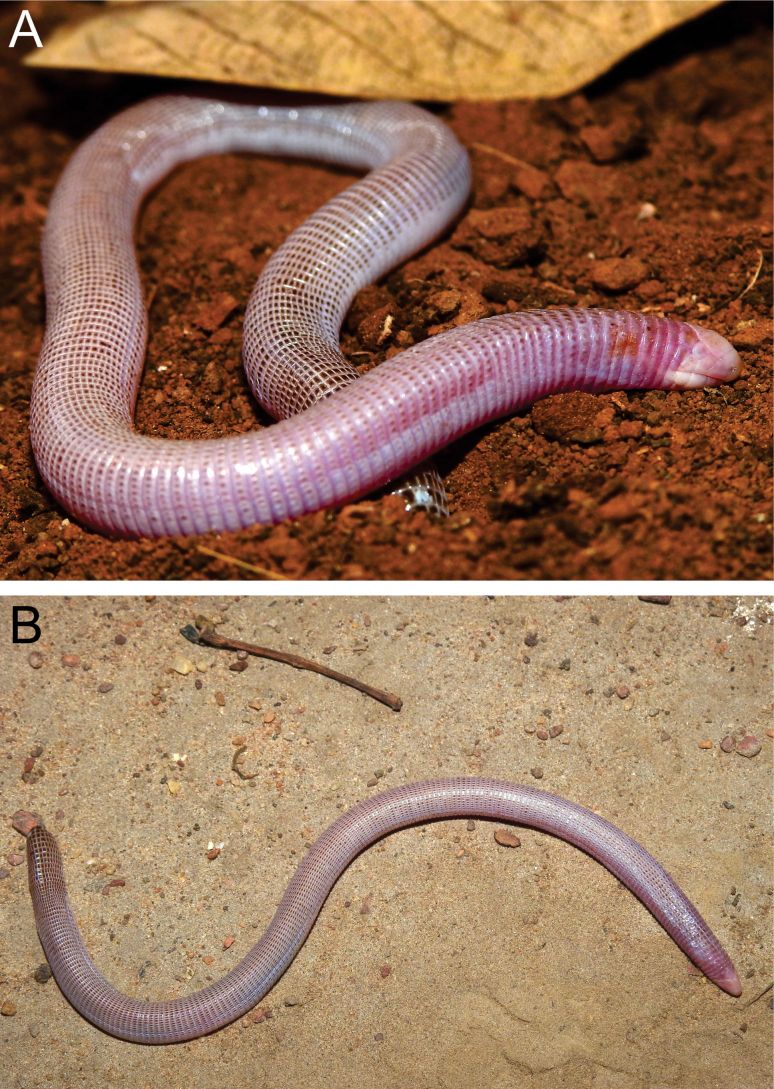
*Amphisbaenaamethysta* sp. nov. in life (not identified specimen of type series) **A** lateral view and **B** dorsal. Photograph by T.B.S.

The main variations in the type series for meristic and morphometric data are given in Table 2. Variation in the arrangement and contact of shields were also observed. CEPB 2280 presents frontal fused with the parietal and segments of first and second body annuli (Fig. 3A), CEPB 2309 present four parietals shields (Fig. 3B), and CEPB 2303 present supralabials fused in left side (Fig. 3C).

**Table 2.** Variation in meristic and morphometric (mm) data in the type series of *Amphisbaenaamethysta* sp. nov. S – sex, DA – dorsal half–annuli, LA – lateral half–annuli adjacent to cloacal region, VA – ventral half–annuli, CA – caudal annuli, AS – autotomic site, DS – dorsal segment in midbody, VS – ventral segment in midbody, SCA – segments of fourth caudal annulus, PRCL – precloacal segments, POCL – postcloacal segments, PGE – postgenials, PA – parietals, SVL – snout–vent length, TL – tail length, BW – body width in midbody, bs – brooked specimen, lta – last tail annuli, n/a – non-available data, +n – mutilated tail, * – tail cicatrised, ** – tail not cicatrised, un- unidentified.

Specimens	S	DA	LA	VA	CA	AS	DS	VS	SCA	PRCL	POCL	PGE1	PA	SVL	TL	BW
**CEPB 2311 (Holotype)**	**male**	**194**	**3**	**195**	**14**	**5**	**18**	**18**	**28**	**6**	**11**	**5**	**1/1**	**233**	**21.2**	**8.2**
CEPB 2301 (Paratype)	female	191	3	191	5 + n	lta5**	19	17	29	7	13	5	1/1	206	3.5 + n	6.4
CEPB 2302 (Paratype)	male	194	4	192	15	5	20	18	31	7	13	5	1/1	199	17.8	7.2
CEPB 2303 (Paratype)	female	194	4	197	5 + n	lta5*	17	16	27	6	14	5	1/1	197	8.2 + n	7.0
CEPB 2308 (Paratype)	female	195	4	196	15	5	18	18	28	n/a	13	4	1/1	150	13.0	5.1
CEPB 2327 (Paratype)	female	196	3	196	14	5	20	19	31	6	13	5	1/1	205	16.5	6.0
CEPB 2331 (Paratype)	female	197	4	195	15	5	18	16	30	6	11	5	1/1	175	14.4	5.8
CEPB 2346 (Paratype)	male	195	4	195	14	5	18	18	29	6	14	5	1/1	138	11.3	4.3
CEPB 2379 (Paratype)	male	185	4	189	5 + n	lta5**	19	18	29	6	13	5	1/1	180	5.0 + n	7.0
CEPB 2381 (Paratype)	female	189	4	189	15	4	16	16	24	6	12	5	1/1	165	14.8	5.6
CEPB 2298	female	199	3	199	14	n/a	18	18	30	7	13	5	1/1	215	16.7	7.4
CEPB 2299	female	193	4	193	6+n	lta5*	18	15	29	6	13	4	1/1	186	7.6 + n	5.1
CEPB 2300	male	194	3	195	14	5	18	17	26	6	12	5	1/1	213	15.3	6.1
CEPB 2304	female	190	4	192	14	5	18	n/a	26	6	12	5	1/1	155	11.6	3.5
CEPB 2305	female	192	4	192	15	5	18	16	28	6	13	6	1/1	170	14.8	5.6
CEPB 2306	female	197	4	196	14	4	18	18	28	8	12	5	1/1	145	10.8	4.2
CEPB 2307	male	190	3	190	6 + n	lta6**	18	17	27	6	13	5	2/2	220	6.8 + n	7.9
CEPB 2309	un	196	3	197	6 + n	lta5*	19	16	n/a	6	14	6	1/2	125	7.9 + n	4.5
CEPB 2310	female	193	4	194	14	5	18	18	26	6	11	2	0/1	195	15.7	5.5
CEPB 2312	female	192	3	192	15	5	18	18	30	6	15	3	1/1	190	15.1	6.6
CEPB 2313	female	189	3	190	15	5	18	15	28	6	14	5	1/1	130	11.7	4.2
CEPB 2314	female	191	3	193	14	5	18	16	26	6	13	6	1/1	185	11.9	5.7
CEPB 2315	female	193	3	193	14	5	18	19	27	6	12	5	1/1	177	14.1	5.9
CEPB 2316	female	195	3	195	16	6	20	18	28	7	13	5	1/1	173	15.2	6.4
CEPB 2317	female	195	5	196	5 + n	lta5**	18	n/a	n/a	6	13	5	1/1	180	3.0 + n	5.3
CEPB 2318	female	196	3	195	15	5	20	18	31	6	11	5	1/1	120	16.1	4.3
CEPB 2319	female	186	4	186	5 + n	lta5**	19	18	29	6	12	5	1/1	95	2.0 + n	3.2
CEPB 2320	female	185	4	182	15	5	19	18	26	7	13	2	1/1	92	7.7	2.6
CEPB 2321	female	194	3	192	16	6	18	18	30	6	12	5	1/1	160	14.1	6.0
CEPB 2322	female	196	4	195	5 + n	lta5**	21	18	27	6	11	4	1/1	155	3.7 + n	5.3
CEPB 2323	female	194	4	193	15	5	16	16	24	6	13	2	1/1	112	8.8	3.0
CEPB 2324	male	193	3	193	16	6	19	18	31	6	13	5	1/1	162	14.6	5.7
CEPB 2325	female	193	4	190	14	5	19	18	27	6	11	5	1/1	174	14.2	6.1
CEPB 2326	male	198	3	197	16	6	17	17	28	6	10	5	1/1	195	16.4	6.4
CEPB 2328	female	192	3	194	15	6	18	18	28	6	12	5	1/1	173	15.5	6.5
CEPB 2329	male	195	4	196	15	5	19	18	30	6	13	6	1/1	170	13.0	5.7
CEPB 2330	male	194	4	193	5 + n	lta5**	20	18	30	6	13	5	1/1	178	2.6 + n	6.0
CEPB 2332	male	190 + n	4	190	15	5	18	16	28	6	15	5	1/1	bs	11.1	4.0
CEPB 2333	female	196	3	194	15	n/a	18	17	26	6	10	2	1/1	203	16.9	6.3
CEPB 2334	female	194	3	192	5 + n	5	20	18	30	6	14	6	1/1	190	7.0 + n	7.3
CEPB 2335	female	197	3	197	14	5	20	18	31	6	12	5	1/1	135	9.7	3.9
CEPB 2336	female	190	4	190	15	6	18	17	29	6	13	4	1/1	105	9.1	3.4
CEPB 2337	female	186	4	185	5 + n	lta5**	19	18	29	6	13	5	1/1	145	3.7 + n	4.9
CEPB 2338	female	190 + n	4	186	13	5	18	17	28	6	12	5	1/1	~ 160	13.8	5.1
CEPB 2339	female	192	4	193	16	5	19	18	32	6	13	5	1/1	127	10.6	4.0
CEPB 2356	male	188	3	189	16	n/a	18	18	28	7	13	3	1/1	190	16.7	5.8
CEPB 2380	female	192	3	195	14	5	17	16	25	6	12	5	2/2	140	11.5	5.6
Minimum		185	3	182	13	4	16	15	24	6	10	2		92	2.2	2.6
Maximum		199	5	199	16	6	21	19	32	8	15	6	n/a	233	21.2	8.2
Mean		192.9	3.6	193.0	14.8	5.0	18.4	17.4	28.3	6.2	12.6	4.7	n/a	167.3	13.4	5.5
Mode		194	4	195	15	5	18	18	28	6	13	5	n/a	190	n/a	n/a
Standard deviation		3.4	0.5	3.4	0.8	0.4	1.0	1.0	1.9	0.5	1.1	1.0	n/a	34.1	3.5	1.3

#### Colour in life.

**Figure 4.**
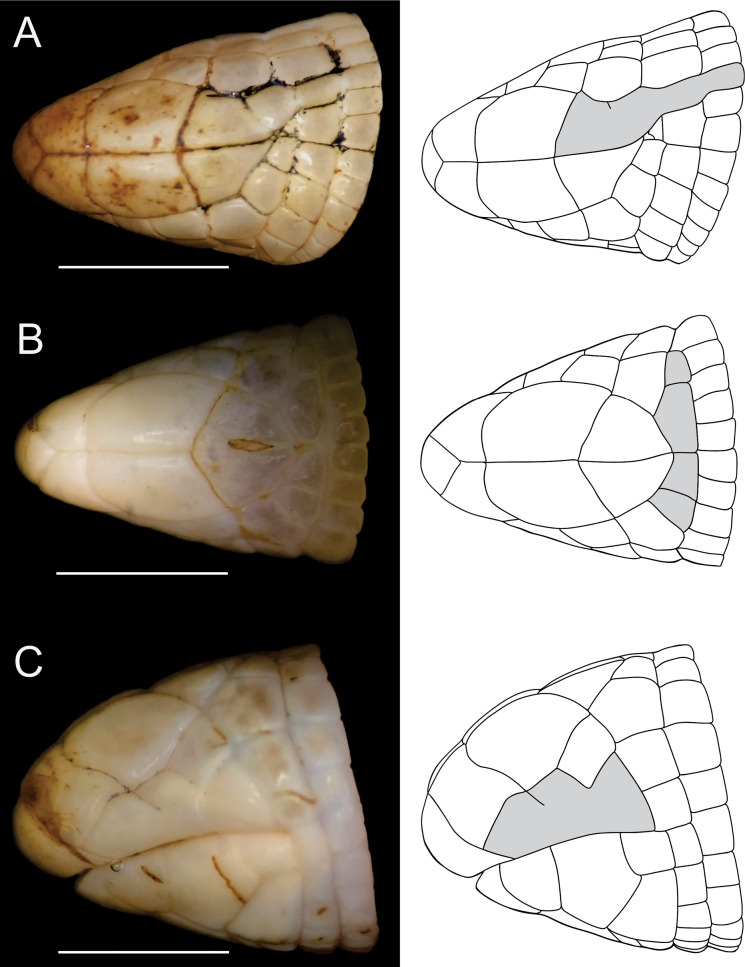
Variation (grey in the drawings) of cephalic shields of *Amphisbaenaamethysta* sp. nov. **A** dorsal view of the head of CEPB 2280 with frontal variation **B** dorsal view of CEPB 2309 with four parietal shields, and **C** lateral view of left side of CEPB 2303 (paratype) with supralabials fused. Scale bar: 3 mm.

Dorsum and lateral parts with dark brown coloration on the segments, which is more pronounced in the vertebral (Fig. 4A) and dorsal section of the tail regions (Fig. 4B). Pink predominates in areas where the brown colour is less pronounced. We do not have photographs of the ventral region of the live specimen.

#### Colour in preservative

**(ethylic alcohol 70%).** Dorsum cream, with brown colouring on the segments in the dorsum, lateral parts, and dorsal portions of the tail portions. Dorsal, lateral, and ventral portions of head pale brown, darker than the ventral portion. Venter cream coloured.

#### Etymology.

The specific epithet refers to the mineral amethyst that is a type of quartz and also the name of the region of the type locality “Brejinho das Ametistas”, a district located south of the municipality of Caetité, state of Bahia. This region has been an amethyst mining centre since the beginning of the 20^th^ century. Spix and Martius (1938) defined the mineral from the “Brejinho das Ametistas” mines as “the beautiful amethysts” on their trip through the “Alto Sertão” of Bahia at the beginning of the 19^th^ century (Cotrim 2015). The region currently has an economy based on mining and energy activities focused on wind energy production. The type series was collected during the execution of environmental programs within the scope of Bahia Mineração (BAMIN), which operates in the exploration of iron ore in the “Brejinho das Ametistas” region.

#### Distribution and habitat.

**Figure 5.**
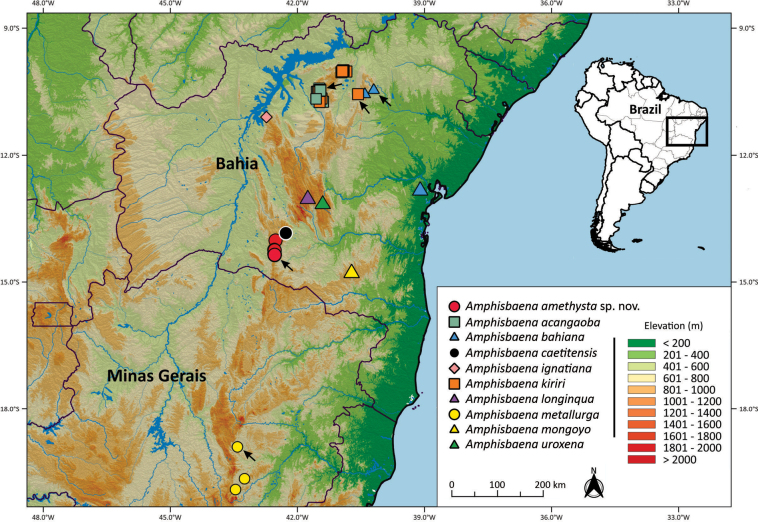
Geographic distribution of *Amphisbaenaamethysta* sp. nov. and others *Amphisbaena* species from Espinhaço Mountain Range locality. Black arrows indicate the type localities of the species with more than one known geographic record.

**Figure 6.**
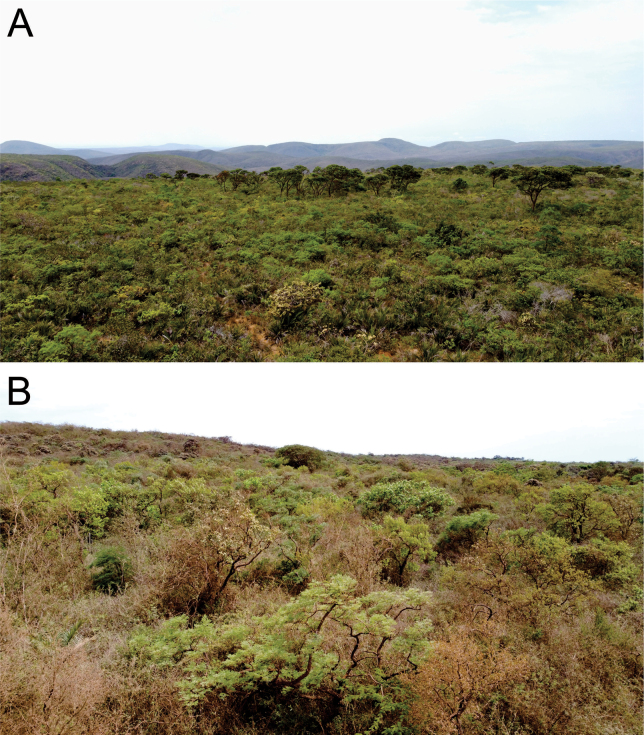
Caatinga site where the holotype of *Amphisbaenaamethysta* sp. nov. was collected in the in the northern portion of Espinhaço Mountain Range, in state of Bahia, Brazil.

*Amphisbaenaamethysta* sp. nov. is known from municipality of Caetité municipality, state of Bahia, Brazil (Fig. 5). The region is in the northern portion of the Espinhaço Mountain Range, has an average altitude of 1000 m a.s.l., and lies within the ecotone between two morphoclimatic domains, Caatinga and Cerrado. In the region there are patches of deciduous and semi-deciduous forests [“Floresta Estacional Decidual” and “Floresta Semidecidual Montana” sensu IBGE (2023)] associated with valleys, slopes, and gallery forests, and containing floristic elements common to the vegetation of the Caatinga, Cerrado, and Atlantic Forest morphoclimatic domains. Areas of savannah vegetation with rock outcrops, typical of the woody Caatinga, occur at higher elevations (Fig. 6).

#### Phylogenetic relationships.

**Figure 7.**
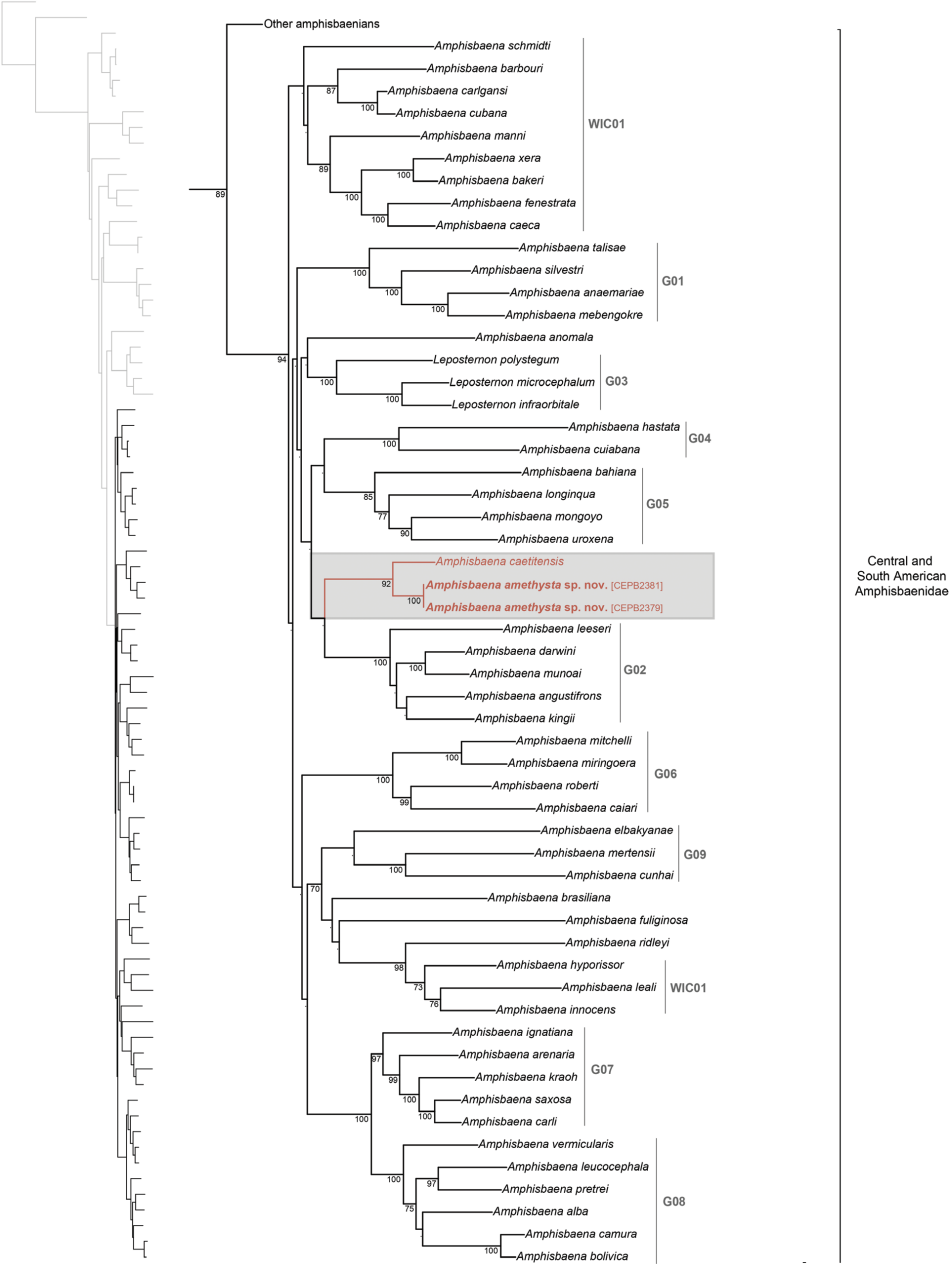
Maximum likelihood tree zoomed in Central and South American Amphisbaenidae resulting from the RAxML analysis based on six concatenated genes, three nuclear (BDNF, c-mos, and RAG1), and three mitochondrial (12S, 16S, and ND2) genes. Red branches denote the clade composed of the new species and its sister species. Numbers on branches represent bootstrap values RAxML (> 70%). The grey codes on the right side of the clades represent the South American subclades of Amphisbaena (G01–G09) and West Indies clades (WIC01–WIC02).

Our concatenated alignment totalled 4806 base pairs (1007 bp for 12S, 528 bp for 16S, 761 bp for *nd2*, 679 bp for *bdnf*, 574 bp for *c-mos*, and 1257 bp for *rag1*). Partition Finder identified a best-fit scheme composed of ten partitions with the GTR + G model. The resulting ML topology (Fig. 7) for the higher-level affinities was similar to those reported by previous studies (Mott and Vieites 2009; Longrich et al. 2015; Graboski et al. 2022) (Fig. 7). *Amphisbaenaamethysta* sp. nov. was recovered as a sister group of *A.caetitensis*, with 92% of bootstrap support. The clade composed by *Amphisbaenaamethysta* sp. nov. and *A.caetitensis* was recovered as a sister group of *A.angustifrons*, *A.darwini*, *A.kingi*, *A.leeseri*, and *A.munoai* with low bootstrap support (21%). The genetic distance (*p-distance*) between *Amphisbaenaamethysta* sp. nov. and *A.caetitensis* is 6.1% for 16S.

## ﻿Discussion

*Amphisbaenaamethysta* sp. nov. is the 73^rd^ species of the genus with four precloacal pores, the 22^nd^ species from Caatinga, and sixth species with a restricted distribution from this morphoclimatic domain. The recognition of *A.amethysta* as a new species is based on molecular data and on set of morphological characters that includes four precloacal pores, a slightly compressed snout, 185–199 dorsal and 182–199 ventral half-annuli, 13–16 caudal annuli, 16–21 dorsal and ventral segments in the midbody, 3/3 supralabials and infralabials, a smooth and rounded tail tip, an autotomic site between 4^th^ and 6^th^ caudal annuli, and by the absence of fusion of the cephalic shields, postmalar shields, and of dorsal and ventral grooves.

The new species is most closely related to *A.caetitensis* (from an elevation of 854 m in the municipality of Caetité, state of Bahia), a sister species with a genetic distance (*p* distance) of 6.1% for the 16S gene, and which differs morphologically mainly in the modified conic pointed tubercules on the tip tail and absence of an autotomic site (see diagnosis). This tail shape appears to have arisen independently in *A.caetitensis* and *A.uroxena*. Almeida et al. (2018) reported a genetic distance of the 7.65% between *A.uroxena* and *A.caetitensis*, and stated that Bayesian inference does not allow a clear resolution of the relationship between the two species. Our phylogenetic analyses corroborate the results of Almeida et al. (2018), and do not structure a clade for species with the modified conic pointed tubercules on the tip tail. We found an even lower distance between *A.caetitensis* and *A.amethysta* sp. nov. and a phylogenetic correlation between the species (Fig. 7), and recovered *A.uroxena* with sister species of *A.mongoyo*, structuring a clade with *A.longinqua* and *A.bahiana* (Clade G05, Fig. 7). Additionally, with a low support, we retrieved the clade formed by *Amphisbaenaamethysta* sp. nov. and *A.caetitensis* as a sister group to clade G02 (Fig. 7) formed by *A.leeseri*, *A.darwini*, *A.munoai*, *A.angustifrons*, and *A.kingi*. This grouping showed a relationship between the species from the Espinhaço Mountain Range and those in other areas of the Atlantic Forest and Cerrado. Additionally, it can indicate a probably convergent evolution between the species with a tuberculate tail shield (*A.caetitensis* and *A.uroxena*) and those species present on mountain within the Espinhaço Mountain Range (clade 5; Fig. 7).

*Amphisbaenaamethysta* appears to be endemic to the northern portion of the Espinhaço Mountain Range with an average altitude of 1000 m a.s.l. Within this, its known extent of occurrence is some 38 km. This distributional pattern is similar to other five species of *Amphisbaena* restricted to the high-altitude areas of the Espinhaço Mountain Range in Bahia [*A.bahiana*, *A.longinqua*, *A.metallurga*, *A.mon­goyo*, and *A.uroxena* (Costa et al. 2015; Teixeira Junior et al. 2019)], four of which are closely related phylogenetically, but show no close phylogenetic relations with *A.amethysta* (*A.metallurga* has no molecular sample available) (Fig. 7). The other three species show no apparent relationship to those from the Espinhaço Mountain Range but also occur at similar altitudes and vegetation types: *A.kiriri* Ribeiro, Gomides & Costa, 2018 (at least 17 km from the nearest *A.bahiana* locality), *A.acangaoba* (occurring in sympatry with *A.kiriri*), and *A.ignatiana* (recorded from the lower sections of the northwestern extremity of the Espinhaço Mountain Range) (Fig. 5) (Roberto et al. 2014; Ribeiro et al. 2018b, 2020).

In the last years morphological characters were commonly used to diagnose *Amphisbaena*, mainly meristics (e.g. Roberto et al. 2014; Costa et al. 2015; Ribeiro et al. 2016, 2019, 2018a, b, 2020; Torres-Ramírez et al. 2021; Ribeiro-Júnior et al. 2022; Rocha et al. 2023) and morphometrics (e.g. Ribeiro et al. 2009; Oliveira et al. 2018) but also with the addition of genetic analyses (e.g., Almeida et al. 2018; Perez and Borges-Martins 2019; Ribeiro et al. 2019), but it is still unclear which characters best represent the interspecific variation of the genus. The identification of diagnostic characters depends on the recognition of intraspecific variations of different species, as well as the analysis of relatively large numbers of samples for each species, the latter being hampered by the fossorial habits of the species in this group and the consequently low frequency of encounters with them. Cryptic species of *Amphisbaena* are rare in bibliographic citations, but recent studies have described new species using diagnoses based on overlapping phenotypic variation and the divergence of the 16S and ND2 genes, complicating species identification using external morphology alone. To reduce the limitations in the identification of new amphisbaenid species, in addition to applying integrative taxonomy whenever possible, we consider it important to standardise the use of meristic and morphometrics characters that do not overlap and to explore new characters, such as the morphometry of the shields (Ribeiro et al. 2008, 2009, 2011, 2015, 2016, 2018a, 2019; Sindaco et al. 2014); analysing characters of internal morphology, such as osteology and gonadal morphology (Ribeiro et al. 2015, 2018a; Oliveira et al. 2018; Angiolani-Larrea et al. 2021); and studying similar species, including type series (Costa et al. 2019; Ribeiro-Júnior et al. 2022).

*Amphisbaenaamethysta* sp. nov. varies in the number of dorsal and ventral half-annuli, caudal annuli, and dorsal and ventral segments in the midbody, the shape and number of parietal shields, and the position of the autotomic site. In addition to variations in shape and number of cephalic shields, specimens of the new species also included individuals with shield fusion, mainly in the parietal region (see discussion of variation). On the other hand, the sample did not vary the number and shape of supra and infralabials and postlabials; the presence of a malar; the shape of the rostral, prefrontal, and frontal shields; the presence and number of precloacal pores; nor in the shape of the segments of the half-annuli of the body and tail. Despite its use as an invariable character, the number of pores can vary intraspecifically. Among the other *Amphisbaena* species with four precloacal pores, 14 vary in the number of precloacal pores: *A.acangaoba*, *A.alba*, *A.angustifrons*, *A.barbouri*, *A.bolivica*, *A.caeca*, *A.camura*, *A.cubana*, *A.darwini*, *A.frontalis*, *A.kraoh*, *A.lumbricalis*, *A.manni*, and *A.uroxena* (Gans 1962a, 1965a; Gans and Alexander 1962; Thomas 1966; Gans and Diefenbach 1972; Hoogmoed and Ávila-Pires 1991; Montero 1996; Vanzolini 1996; Mott et al. 2008; Perez et al. 2012; Ribeiro et al. 2020; Ribeiro-Júnior et al. 2022). Additionally, sexual dimorphism in the presence or absence of pores for *Amphisbaena* has already been reported for *A.frontalis* and *A.uroxena* (Mott et al. 2011; Ribeiro-Júnior et al. 2022).

Mining activities cause several irreversible changes to the environment, including the loss of habitat due to the removal of vegetation and the relocation and excavation of soil during the opening and operation of new mines. Such actions have a direct impact on terrestrial and fossorial species such as amphisbaenians. In this context, applied studies, such as the evaluation of species as biomarkers for metal exposure, mainly with fossorial reptile species, are neglected (Grillitsch and Schiesari 2010; Gil-Jiménez et al. 2021). In such circumstances, the development and execution of environmental programs are critical for effective impact mitigation. Such studies can form valuable tools when conducted by specialised teams with a focus on the scientific use of collected material (Vaz-Silva 2009), and teams concerned with the technical quality of the project, which would result in more careful projects in relation to the sampling design and analytical aspects of the results, allowing greater decision-making power for effective conservation actions (Dias et al. 2019). In this case, environmental studies have made an important contribution to knowledge of the biodiversity of the area under BAMIN’s control, Bahia Mineração. Finally, the identification of a new species indicates that the fossorial fauna, as well as that of other groups, in the Espinhaço Mountain Range region is far from being completely known and that it may harbour a much greater diversity of endemic taxa than has been realised so far.

## Supplementary Material

XML Treatment for Amphisbaena amethysta

## ﻿Additional information

### Author ORCIDs

Síria Ribeiro https://orcid.org/0000-0002-2301-7089

Alfredo P. Santos Jr https://orcid.org/0000-0002-2829-718X

Isabelly G. Martins https://orcid.org/0009-0002-4420-5713

Roberta Graboski https://orcid.org/0000-0002-9123-4819

Wilian Vaz-Silva https://orcid.org/0000-0001-6235-5331

### Data availability

All of the data that support the findings of this study are available in the main text or Supplementary Information.

## Supplementary materials

Supplementary material 1 Supplementary information Data type docx https://binary.pensoft.net/file/1140649This dataset is made available under the Open Database License (http://opendatacommons.org/licenses/odbl/1.0/). The Open Database License (ODbL) is a license agreement intended to allow users to freely share, modify, and use this Dataset while maintaining this same freedom for others, provided that the original source and author(s) are credited.Síria Ribeiro, Alfredo P. Santos Jr, Isabelly G. Martins, Elaine C. S. Oliveira, Roberta Graboski, Thiago Barbosa Da Silveira, Matheus H. M. Benício, Wilian Vaz-Silva
