# Supplementary material for: ﻿A new four-pored Amphisbaena Linnaeus, 1758 (Amphisbaenia, Amphisbaenidae) from the north of Espinhaço Mountain Range, Brazil
Source: Zookeys. 2024 Sep 24;1213:1–27. doi: 10.3897/zookeys.1213.122265 (PMC11445610; doi:10.3897/zookeys.1213.122265)
Supplement: Supplementary material 1 — Supplementary information [file zookeys-1213-001_article-122265__-s001.docx]

**Supplementary information 1.** Collections consulted and specimens used in the comparative analysis.

Scientific collections (acronyms are presented according to the designations of the institutions): AMNH_ Herpetological of the American Museum of Natural History; ANSP_ Academy of Natural Sciences Philadelphia (ANSP); CEPB–PUC Goiás_ Coleção Herpetologica da Pontifícia Universidade Católica de Goiás, Goiânia, Goiás, Brazil; CEUCH_ Coleção Zoológica de Referência do Campus de Corumbá da Universidade Federal de Mato Grosso do Sul, Campo Grande, Mato Grosso do Sul, Brazil; CHUNB_ Herpetológica da Universidade de Brasília, Brasília, Brazil; LPHA_ Linha de Pesquisa em Herpetologia da Amazônia of Faculdades Integradas do Tapajós, Santarém, Pará, Brazil; MCP_ Museu de Ciências e Tecnologia da Pontifícia Universidade Católica do Rio Grande do Sul, Porto Alegre, Rio Grande do Sul, Brazil; MNHNP_ Museo Nacional de Historia Natural del Paraguay, San Lorenzo, Paraguay; MNRJ_ Museu Nacional, Rio de Janeiro, Rio de Janeiro, Rio de Janeiro, Brazil; MPEG_ Museu Paraense Emílio Goeldi, Belém, Pará, Brazil; MSNG_ Museo Civico di Storia Naturale “Giacomo Doria” of Genoa; MUFAL_ Museu de História Natural da Universidade Federal de Alagoas, Maceió, Alagoas; MZUSP_ Museu de Zoologia da Universidade de São Paulo, São Paulo, São Paulo, Brazil; UFMT_ Coleção Zoológica de Vertebrados da Universidade Federal do Mato Grosso, Cuiabá, Mato Grosso, Brazil; UFOPA-H_ Coleção Herpetológica da Universidade Federal do Oeste do Pará, Santarém, Pará, Brazil; ZUEC_ Coleção de répteis do Museu de Zoologia da UNICAMP; ZUFG_ Coleção Zoológica da Universidade Federal de Goiás.

*Amphisbaena alba* (*n =* 5). BRAZIL: CEUCH 1851. MATO GROSSO DO SUL: Corumbá: CEUCH 1731, CEUCH 1847, CEUCH 3553. BAHIA: Camaçari: MCP 18177.

*Amphisbaena acrobeles* (*n =* 1). BRAZIL: TOCANTINS: Jalapão: MZUSP 96337 (Holotype).

*Amphisbaena anaemariae* (*n =* 6). BRAZIL: GOIÁS: Campinaçu: MZUSP 103743; São Salvador do Tocantins, UHE São Salvador: MZUSP 99394; UHE Cana Brava: MZUSP 97217; UHE Serra da Mesa: MZUSP 80596 (Holotype), MZUSP 97047, MZUSP 97171.

*Amphisbaena anomala* (*n =* 42). BRAZIL: MPEG 27922–27924, MPEG 28550, UFOPA (L 2273, L 564, L 563, B 67); CEARÁ: Cinta Boa Vista: MNRJ 9313–9314; MARANHÃO: Nova Vida: MPEG 9942–9943, MPEG 12715–12716; Paruá: MPEG 10527, MPEG 11498; PARÁ: Ananindeua: MPEG 222, MPEG 4550; Baião: MPEG 15715; Belém: MNRJ 1772, MPEG 12873, MPEG 10722–10725; Benevides: MPEG 9925; Bragança: MPEG 6565, MPEG 9200; Castanhal: MPEG 7239, MPEG 8720; Capitão Poço: MPEG 7257, MPEG 8033; Curuçá: MPEG 6735; Marabá: MPEG 7264; Igarapé-Açú: MPEG 22231; Irituia: MPEG 30049; Km 224 da BR 316: MPEG 10741; Ourém: MPEG 5292, MPEG 22359 (Patauateua); Paragominas: MPEG 10467; Peixe-boi: MPEG 6057; Viseu: MPEG 9438.

*Amphisbaena arda* (*n =* 1). BRAZIL: BAHIA: Mocambo do Vento: MZUSP 91638 (Holotype).

*Amphisbaena arenaria* (*n =* 1). BRAZIL: BAHIA: Raso da Catarina: MZUSP 65817 (Holotype).

*Amphisbaena bahiana* (*n =* 1). BRAZIL: BAHIA: Senhor do Bonfim (= Vila Nova da Rainha): MZUSP 1259 (Holotype).

*Amphisbaena bedai* (*n =* 8). BRAZIL: MATO GROSSO DO SUL: Anastácio: MZUSP 72988 (Holotype); Guia Lopes da Laguna: MZUSP 73069 (Paratype); Aquidauana: MCP 8923–8924, AFB 783, AFB 2137, AFB 2143, AFB 3138.

*Amphisbaena bilabialata* (*n =* 5). BRAZIL: MATO GROSSO: Nova Ubiratã: UFMT 4772–4773; Cláudia*:* MZUSP 81777; Xavantina: MZUSP 21276 (Holotype), MZUSP 21777 (Paratype).

*Amphisbaena borellii* (*n =* 1). PARAGUAI: MNHNP 5103.

*Amphisbaena brasiliana* (*n =* 70). BRAZIL: PARÁ: Aveiro: LPHA 1264; Juruti: MPEG 25288–25291; Marabá: MPEG 12932; Jacareacanga: UFMT 8477; Santarém: LPHA 107–109, LPHA 111–114, LPHA 116, LPHA 238, LPHA 339, LPHA 416, LPHA 623, LPHA 890–891, LPHA 895, LPHA 897–898, LPHA 900, LPHA 1039, LPHA 1110, LPHA 1202–1203, LPHA 1220, LPHA 1286–1287, LPHA 1294–1296, LPHA 1351, LPHA 1372, LPHA 1541, LPHA 1543, LPHA 1654, LPHA 1703, LPHA 2574–2575, LPHA 2802, LPHA 2839, LPHA 2900, LPHA 2989, LPHA 2990, LPHA 3047, LPHA 3595, LPHA 4360–4362, LPHA 4604, LPHA 5152–5154, UFOPA-H 115, UFOPA-H 217, UFOPA-H 224, UFOPA-H 396, UFOPA-H 519, UFOPA-H 530, UFOPA-H 546–547, UFOPA-H 571, UFOPA-H 575, UFOPA-H 875–877; MATO GROSSO: Alta Floresta: UFMT 7785.

*Amphisbaena carli* (*n =* 2). BRAZIL: BAHIA: Jaborandi: MZUSP 96346 (Holotype – MNRJ 19256); MZUSP 96345 (Paratype).

*Amphisbaena crisae* (*n =* 3). BRAZIL: MATO GROSSO: Barra do Tapirapé: MZUSP 6418 (Holotype), MZUSP 6417 (Paratype). MNRJ 12462.

*Amphisbaena cuiabana* (*n =* 10). BRAZIL: ZUEC 2083–2091; MATO GROSSO: Cuiabá, Chapada dos Guimarães: CHUNB 13652.

*Amphisbaena cunhai* (*n =* 5). BRAZIL: RONDÔNIA: Porto Velho: MZUSP 101719, MZUSP 101721, MZUSP 101734, MZUSP 102237. AMAZONAS: Rio Tuxi: MPEG 20373.

*Amphisbaena darwini* (*n =* 7). BRAZIL: RIO GRANDE DO SUL: Dom Pedro de Alcântara: MCP 3618; Viamão: MCP 5613–5615; SANTA CATARINA: Lauro Müller, Novo Horizonte: MZUSP 21467; URUGUAY: Montevideo: Puntas de Manga: MZUSP 82343–82344.

*Amphisbaena frontalis* (*n =* 17). BRAZIL: BAHIA: Alagoado: MZUSP 72989 (Holotype); MZUSP 18112, MZUSP 96997. MARANHÃO: MPEG 27925–27932; Urbano Santos: MZUSP 91989 (Holotype of former *A*. *ibijara*), MZUSP 99302, MZUSP 99304–99306, MZUSP 99328.

*Amphisbaena fuliginosa* (*n =* 6). BRAZIL: CHUNB 49941, 47715, MNRJ 8224, AMNH R 37442; AMAZONAS: Manaus: AMNH R 64919. ECUADOR: Chimborazo: AMNH R 23324. GUYANA: Mazaruni-Potaro: AMNH R 137422.

*Amphisbaena hogei* (*n =* 1). BRAZIL: SÃO PAULO: Ilha dos Alcatrazes: MZUSP 6905 (Holotype).

*Amphisbaena hoogmoedi* (*n =* 5). BRAZIL: PARÁ: Jacareacanga – Teles Pires hydroelectric dam: MPEG 32283–85, MZUSP 106219 (Holotype), MZUSP 106220.

*Amphisbaena ignatiana* (*n =* 1). BRAZIL: BAHIA: Santo Inácio: MZUSP 72616 (Holotype).

*Amphisbaena kingi* (*n =* 13). BRAZIL: RIO GRANDE DO SUL: MCP 15052, MCP 15056–15057, MCP 15106, MCP 15403, MCP 15407–15408, MCP 16181, MCP 16186, MCP 16724, MCP 17708, MCP 17769, MCP 17771.

*Amphisbaena kraoh* (*n =* 7). BRAZIL: TOCANTINS: Pedro Afonso: MZUSP 2520 (Holotype); *Mateiros*: CHUNB 30667; MARANHÃO: Estreito: MZUSP 099299–099301, MZUSP 099307–099308.

*Amphisbaena leeseri* (*n =* 3). BRAZIL: MATO GROSSO: Aquidauana: MZUSP 82539–82540; Guia Lopes da Lagoa: MZUSP 73313.

*Amphisbaena lumbricalis* (*n =* 26). BRAZIL: SERGIPE: MUFAL 2777–2784, MUFAL 2789, MUFAL 2790, MUFAL 2796–2798, MUFAL 2800, MUFAL 2806, UFAL 2816, MMUFAL 9071, MUFAL 9810, MZUSP 79420–79422, MZUSP 79424–79428 (Paratype).

*Amphisbaena mebengokre* (*n =* 12). BRAZIL: GOIÁS: Hydroelectric Power Plant Santo Antônio do Caiapó, Arenópolis: MNRJ 25189 (Holotype), MNRJ 25191, MNRJ 25194, MNRJ 25196, MNRJ 25198, MPEG 32206, ZUFG 1194, ZUFG 1196, ZUFG 1201, ZUFG 1204, ZUFG 1205, ZUFG 1207.

*Amphisbaena mertensi* (*n =* 1). BRAZIL: GOIÁS: Aporé: MNRJ 14117.

*Amphisbaena miringoera* (*n =* 1). BRAZIL: MATO GROSSO: Porto Velho: Rio Tapirapés: MZUSP 13756 (Holotype).

*Amphisbaena mitchelli* (*n =* 9). BRAZIL: MARANHÃO: Santa Luzia do Paruá: MPEG 11205, MPEG 11209; Puraquéu: MPEG 11759; PARÁ: Barcarena: MPEG 28816; Carajás: MPEG 13993, MPEG 13996; Belterra: LPHA 4942; Belém: MZUSP 7140; Juruá, Rio Xingu: MZUSP 67714.

*Amphisbaena munoai* (*n =* 4) BRAZIL: RIO GRANDE DO SUL: Guaporé: MCP 15617–15620.

*Amphisbaena neglecta* (*n =* 3): BRAZIL: Mato Grosso: Chapada dos Guimarães: ANSP 1309 (Holotype, by photography), ANSP 13020-13021 (Paratypes, by photography).

*Amphisbaena nigricauda* (*n =* 1). BRAZIL: MNRJ 2102.

*Amphisbaena pretrei* (*n =* 1). BRAZIL: RIO GRANDE DO NORTE: Natal: MPEG 30168.

*Amphisbaena prunicolor* (*n =* 5). BRAZIL: RIO GRANDE DO SUL: Bento Gonçalves: MCP 963; Dom Pedro de Alcântara: MCP 1544; Porto Alegre: MCP 341–342, MCP 1551.

*Amphisbaena roberti* (*n =* 22). BRAZIL: SÃO PAULO: EE Águas de Santa Bárbara: UFOPA-H 3371, UFOPA-H 3372 (former CN 1001, CN 1014); Ipiranga: MZUSP 1946 (Holotype), MZUSP 1257 (Paratype); Marília: UFMT 4713; MATO GROSSO: Chapada dos Guimarães: UFMT 362–363, UFMT 6122; GÓIAS: UFOPA-H 3360–3369 (former RE 24, RE 46, RE 59, RE 83, RE 242, RE 339, RE 1946, RE 2100, RE 3125, RE 7624); Aporé: UFOPA-H 869–871; MATO GROSSO DO SUL: Sonora: UFOPA 3370 (former RE 463).

*Amphisbaena sanctaeritae* (*n =* 1). BRAZIL: SÃO PAULO: Santa Rita do Passo Quatro: MZUSP 36719 (Holotype).

*Amphisbaena saxosa* (*n =* 20). BRAZIL: TOCANTINS: UHE de Peixe Angical: MZUSP 96651– 96659; Lajeado – UHE Luís Eduardo Magalhães: MZUSP 91546 (Holotype); MZUSP 95143–95152.

*Amphisbaena silvestrii* (*n =* 9). BRAZIL: MNRJ 12457; MATO GROSSO: Querência: MPEG 24489; Nobres: CHUNB 40780; Araputanga UFMT 2676, UFMT 2680, UFMT 2699; Barra do Garças: UFMT 2616; Campo Novo do Parecis: UFMT 4752; Cuiabá: MSNG 28308 (Holotype, by photography).

*Amphisbaena steindachneri* (*n =* 18). BRAZIL: MATO GROSSO: Campo Novo do Parecis: 6029; Jauru: UFMT 3345, UFMT 3347; Tangará da Serra: UFMT 7554; UFMT 7555; Juscimeira: UFMT 8860, UFMT 8861, UFMT 8951–8952; Santo Antônio do Leverger: UFMT 6610–6611, UFMT 6613–6614; Tangará da Serra: UFMT 7554–7555; MATO GROSSO DO SUL: Corumbá: UFMT 2487; RONDÔNIA: Pimenta Bueno: CHUNB 18373, CHUNB 18378.

*Amphisbaena talisiae* (*n =* 3). BRAZIL: GOIÁS: Chapada dos Veadeiros: MNRJ 4794; Serra da Mesa: MZUSP 83231, MZUSP 83234 (Holotype and paratype of former *A*. *mensae*, respectively).

*Amphisbaena tragorrhectes* (*n =* 1). BRAZIL: PARÁ: Oriximiná: MZUSP 17518 (Holotype).

*Amphisbaena vanzolinii* (*n =* 2). BRAZIL: AMAZONAS: MPEG 14804; Balbina: MZUSP 68644 (Holotype of former *A*. *hugoi*).

*Amphisbaena vermicularis* (*n =* 8). BRAZIL: PIAUÍ: Serra das Confusões: MZUSP 97991; Maranhão: MPEG 27933–27935, MPEG 27937, MPEG 28542–28544.

| **Table S1:** List of taxa and GenBank accession number of specimens used for this study. | | | | | | |
| --- | --- | --- | --- | --- | --- | --- |
| **ID** | **12S** | **16S** | ***nd2*** | ***c-mos*** | ***rag-1*** | ***bdnf*** |
| *Agamodon anguliceps* | - | - | - | AY44401.3 | AY44404.0 | - |
| *Amphisbaena alba* | EU20364.4 | FJ44170.4 | FJ44194.7 | FJ44176.4 | FJ44182.4 | FJ441887 |
| *Amphisbaena amethysta* sp. nov. CEPB2379 | PQ27677.4 | PQ276772 |  |  |  |  |
| *Amphisbaena amethysta* sp. nov. CEPB2381 | PQ27677.5 | PQ276773 |  |  |  |  |
| *Amphisbaena anaemariae* | - | FJ441668 | FJ441911 | FJ441728 | FJ441788 | FJ441851 |
| *Amphisbaena angustifrons* | - | FJ441707 | FJ441950 | FJ441767 | FJ441827 | FJ441890 |
| *Amphisbaena anomala* | - | FJ441712 | FJ441955 | FJ441772 | FJ441832 | FJ441895 |
| *Amphisbaena arenaria* | - | KY018688 | KY018695 | KY018693 | - | KY018691 |
| *Amphisbaena bahiana* | - | MG028546 | MG028575 | MG028566 | MG028585 | MG028557 |
| *Amphisbaena bakeri* | EU20364.5 | MH311045 | - | - | - | - |
| *Amphisbaena barbouri* | EU20364.6 | EU203646 | - | - | - | - |
| *Amphisbaena bolivica* | - | FJ441669 | FJ441912 | FJ441729 | FJ441789 | FJ441852 |
| *Amphisbaena brasiliana* |  | FJ441708 | FJ441951 | FJ441768 | FJ441828 | FJ441891 |
| *Amphisbaena caeca* | EU20364.7 | FJ441708 | FJ441914 | FJ441731 | FJ441791 | FJ441854 |
| *Amphisbaena caiari* | - | KJ669327 | KJ669333 | KJ669331 | KJ669335 | KJ669329 |
| *Amphisbaena camura* | - | FJ441672 | FJ441915 | FJ441732 | FJ441792 | FJ441855 |
| *Amphisbaena carlgansi* | EU20364.8 | EU203648 | - | - | - | - |
| *Amphisbaena carli* | - | KY352316 | KY352335 | KY352328 | KY352331 | KY352324 |
| *Amphisbaena cubana* | EU20364.9 | EU203649 | - | AY487346 | EU203632 | EU203609 |
| *Amphisbaena cuiabana* | - | FJ441696 | FJ441939 | FJ441756 | FJ441816 | FJ441879 |
| *Amphisbaena cunhai* | - | FJ441673 | FJ441916 | FJ441733 | FJ441793 | FJ441856 |
| *Amphisbaena darwinii* | - | FJ441693 | FJ441936 | FJ441753 | FJ441813 | FJ441876 |
| *Amphisbaena elbakyanae* | - | - | MT433763 | - | - | - |
| *Amphisbaena fenestrata* | EU20365.0 | EU203650 | - | - | - | - |
| *Amphisbaena fuliginosa* MZUSP82798 | - | FJ441684 | FJ441927 | FJ441744 | FJ441804 | FJ441867 |
| *Amphisbaena hastata* |  | FJ441677 | FJ441920 | FJ441737 | FJ441797 | FJ441860 |
| *Amphisbaena hyporissor* | EU20365.1 | EU203651 | - | - | - | - |
| *Amphisbaena ignatiana* |  | FJ441679 | FJ441922 | FJ441739 | FJ441799 | FJ441862 |
| *Amphisbaena innocens* | EU20365.2 | EU203652 | - | - | - | - |
| *Amphisbaena kingi* | - | FJ441725 | FJ441968 | FJ441785 | FJ441846 | FJ441908 |
| *Amphisbaena kraoh* | - | FJ441692 | FJ441935 | FJ441752 | FJ441812 | FJ441875 |
| *Amphisbaena leali* | EU20365.3 | EU203653 | - | - | - | - |
| *Amphisbaena leeseri* |  | FJ441694 | FJ441937 | FJ441754 | FJ441814 | FJ441877 |
| *Amphisbaena leucocephala* | - | KY352317 | KY352336 | KY352329 | KY352332 | KY352325 |
| *Amphisbaena longinqua* | - | MN713822 | MN713831 | MN713827 | MN713839 | MN713834 |
| *Amphisbaena manni* | EU20365.4 | EU203654 | - | - | - | - |
| *Amphisbaena mensae* | - | - | MN713832 | MN713829 | MN713841 | MN713836 |
| *Amphisbaena mertensi* | - | FJ441674 | FJ441917 | FJ441734 | FJ441794 | FJ441857 |
| *Amphisbaena miringoera* | - | KT371482 | - | KT371478 | - | - |
| *Amphisbaena mitchelli* |  | KY018690 | KY018696 | KY018694 | KY018697 | KY018692 |
| *Amphisbaena mongoyo* | - | MN713826 | MN713833 | MN713830 | MN713843 | MN713838 |
| *Amphisbaena munoai* | - | FJ441687 | FJ441930 | FJ441747 | FJ441807 | FJ441870 |
| *Amphisbaena pretrei* |  | KY352320 | KY352338 | KY352330 | KY352334 | KY352327 |
| *Amphisbaena ridleyi* | OK52333.9 | OK523354 | - | OK523366 | OK523371 | OK523360 |
| *Amphisbaena roberti* | - | MG028547 | MG028576 | MG028567 | MG028586 | MG028558 |
| *Amphisbaena saxosa* |  | FJ441709 | FJ441952 | FJ441769 | FJ441829 | FJ441892 |
| *Amphisbaena schmidti* | EU20365.5 | FJ441681 | FJ441924 | FJ441741 | FJ441801 | FJ441864 |
| *Amphisbaena silvestrii* | - | FJ441688 | FJ441931 | FJ441748 | FJ441808 | FJ441871 |
| *Amphisbaena* sp. | - | FJ441690 | FJ441933 | FJ441750 | FJ441810 | FJ441873 |
| *Amphisbaena* sp. | - | FJ441691 | FJ441934 | FJ441751 | FJ441811 | FJ441874 |
| *Amphisbaena uroxena* |  | MG028550 | MG028578 |  | MG028587 | MG028559 |
| *Amphisbaena vermicularis* |  | MG028551 | MG028579 | MG028568 | MG028588 | MG028560 |
| *Amphisbaena xera* | EU20365.6 | MH311054 | AY662541 | AY662568 | AY662619 | - |
| *Bipes biporus* | - | - | NC6287 | AF039482 | HQ876445 | HM160582 |
| *Bipes canaliculatus* | EU20365.8 | EU203658 | NC6288 | FJ518700 | FJ518701 | FJ441849 |
| *Bipes tridactylus* | - | - | NC6286 | - | - | - |
| *Blanus cinereus* | EU44325.7 | KC621324 | NC12433 | EU116674 | AY444045 | - |
| *Blanus strauchi* | EU20366.0 | FJ518702 | FJ518703 | AY444024 | AY444050 | FJ441847 |
| *Blanus tingitanus* | - | KJ624837 | DQ902269 | DQ324866 | - | - |
| *Cadea blanoides* | EU20366.1 | EU203661 | - | EU203613 | EU203662 | EU203612 |
| *Chirindia swynnertoni* | - | HG425323 | HG425312 | AY444010 | AY444037 | - |
| *Cynisca kraussi* | - | HG425322 | - | HG425295 | HG425277 | - |
| *Cynisca leucura* | - | - | - | AY444011 | AY444038 | - |
| *Dalophia ellenbergeri* | - | HG425315 | HG425304 | HG425299 | HG425283 | - |
| *Dalophia pistillum* | - | HG425320 | HG425313 | HG425294 | HG425282 | - |
| *Dalophia* sp. | - | HG425321 | - | - | HG425279 | - |
| *Diplometodon zarudnyi* | MG66125.4 | MG661255 | NC6283 | AY444023 | JN654858 | JN654798 |
| *Geocalamus acutus* | AB16290.9 | AB162909 | AB162909 | AY444017 | JN654860 | JN654800 |
| *Amphisbaena infraorbitale* |  | FJ441722 | FJ441965 | FJ441782 | FJ441842 | FJ441905 |
| *Amphisbaena microcephalum* |  | FJ441716 | FJ441959 | FJ441776 | FJ441836 | FJ441899 |
| *Amphisbaena polystegum* |  | FJ441721 | FJ441964 | FJ441781 | FJ441841 | FJ441904 |
| *Monopeltis capensis* | - | HG425324 | HG425311 | HG425298 | HG425281 | - |
| *Monopeltis sphenorhynchus* | - | - | HG425309 | HG425296 | HG425276 | - |
| *Rhineura floridana* | KU74459.4 | EU203657 | NC6282 | AY444021 | AY444047 | FJ441848 |
| *Trogonophis wiegmanni* | MG66109.0 | MG661128 | FJ441910 | MG661253 | AY444051 | FJ441850 |
| *Zygaspis maraisi* | - | LT558090 | LT558091 | - | - | - |
| *Zygaspis nigra* | - | HG425317 | HG425310 | HG425293 | HG425288 | - |
| *Zygaspis quadrifrons* | - | HG425316 | HG425307 | HG425292 | HG425287 | - |
| *Zygaspis vandami* | - | HG425314A | HG425302 | HG425289 | HG425284 | - |
| *Zygaspis vandami* | - | - | HG425303 | - | HG42528.5 | - |
